# Deletion of microRNA-80 Activates Dietary Restriction to Extend *C. elegans* Healthspan and Lifespan

**DOI:** 10.1371/journal.pgen.1003737

**Published:** 2013-08-29

**Authors:** Mehul Vora, Mitalie Shah, Silvana Ostafi, Brian Onken, Jian Xue, Julie Zhouli Ni, Sam Gu, Monica Driscoll

**Affiliations:** Department of Molecular Biology and Biochemistry, Nelson Biological Laboratories, Rutgers, The State University of New Jersey, Piscataway, New Jersey, United States of America; Stanford University Medical Center, United States of America

## Abstract

Caloric/dietary restriction (CR/DR) can promote longevity and protect against age-associated disease across species. The molecular mechanisms coordinating food intake with health-promoting metabolism are thus of significant medical interest. We report that conserved *Caenorhabditis elegans* microRNA-80 (*mir-80*) is a major regulator of the DR state. *mir-80* deletion confers system-wide healthy aging, including maintained cardiac-like and skeletal muscle-like function at advanced age, reduced accumulation of lipofuscin, and extended lifespan, coincident with induction of physiological features of DR. *mir-80* expression is generally high under *ad lib* feeding and low under food limitation, with most striking food-sensitive expression changes in posterior intestine. The acetyltransferase transcription co-factor *cbp-1* and interacting transcription factors *daf-16/FOXO* and heat shock factor-1 *hsf-1* are essential for *mir-80*(Δ) benefits. Candidate miR-80 target sequences within the *cbp-1* transcript may confer food-dependent regulation. Under food limitation, lowered miR-80 levels directly or indirectly increase CBP-1 protein levels to engage metabolic loops that promote DR.

## Author Summary

Dietary restriction, limitation of calorie intake with maintained vitamin and mineral support, can extend lifespan and protect against diseases of age across many species. Elaboration of molecular mechanisms that control dietary restriction in simple animal models may therefore inform on strategies to activate health-promoting metabolism to help address clinical challenges associated with aging and age-associated disease. We characterize a single *Caenorhabditis elegans* microRNA gene that keeps dietary restriction programs off when food is abundant. A *mir-80* deletion exhibits beneficial features of dietary restriction regardless of food availability, including extended maintenance of mobility and cardiac-like muscle function later into life as well as lifespan extension. We identify three key longevity genes that are required for these benefits. We hypothesize that miR-80 is a core regulator by which diverse and intersecting metabolic pathways are coordinately regulated to respond to nutrient availability.

## Introduction

The promotion of healthy aging is a goal of modern medicine, and simple interventions that protect against age-associated decline and disease are the dream of many in the general population. Genetics, environment, and stochastic factors all make substantial and complex contributions to healthspan. Single gene mutations that affect conserved pathways in model organisms can extend life and slow age-associated decline [1], [2]. Environmental factors such as diet can also have a profound effect on the quality of aging. For example, dietary restriction (DR), limitation of calorie intake with maintained vitamin and mineral support, can extend lifespan and protect against diseases of age across many species [3]. Elaboration of molecular mechanisms that control DR in simple animal models may thus inform on strategies to activate health-promoting metabolism to help address clinical challenges associated with aging.

In the nematode *Caenorhabditis elegans*, food limitation that results in lifespan extension can be introduced via several protocols [4], [5], [6], [7], [8], although the specific genetic requirements for longevity benefits of different DR regimens are not fully overlapping. For example, the transcription factor DAF-16/FOXO is dispensable for longevity induced in the feeding-impaired *eat-2* mutant, whereas with a DR protocol in which bacterial food is diluted on plates, DAF-16/FOXO is essential for lifespan extension [4], [9]. Such observations most likely reflect highly complex regulatory loops that control the precise metabolic state.

microRNAs (miRNAs) can be metabolic regulators [10]. miRNAs are small, ∼22 nt non-coding RNAs that can bind to transcripts via partial sequence complementarity to down-regulate translation of those target mRNAs. Many miRNAs are conserved over their lengths or in the critical 5′ seed region, defining families across species [11], [12], [13]. Although the co-evolution of miRNAs and their targets is a complex process [14], some miRNA/target pairings have been molecularly and functionally conserved. For example, discovery of LET-7 miRNA regulation of target RAS in *C. elegans* [15] inspired anti-oncogenic therapies for mammalian lung tumors [16].

We took advantage of the powerful reagents for miRNA study in *C. elegans* [17], [18] and our previous characterization of a DR fluorimetric signature of endogenous gut fluorescence in these transparent nematodes (derived from lipofuscin+advanced glycation end products [19]) to identify miRNAs that might regulate DR. Here we report bantam-homolog miR-80 as a food-regulated miRNA that normally represses DR when food is abundant. Transcription factors DAF-16, HSF-1, and CBP-1 are required for *mir-80*(Δ) benefits. Of these, the *cbp-1* transcript includes sequences that might be directly targeted by miR-80 to coordinate this circuit. Our data suggest an approach to metabolic activation of DR even under *ad lib* feeding that could inspire strategies for treating obesity, limiting age-associated disease, and promoting healthy aging.

## Results

### Deletion of microRNA-80 promotes system-wide healthy aging in *C. elegans*

**Figure 1:**
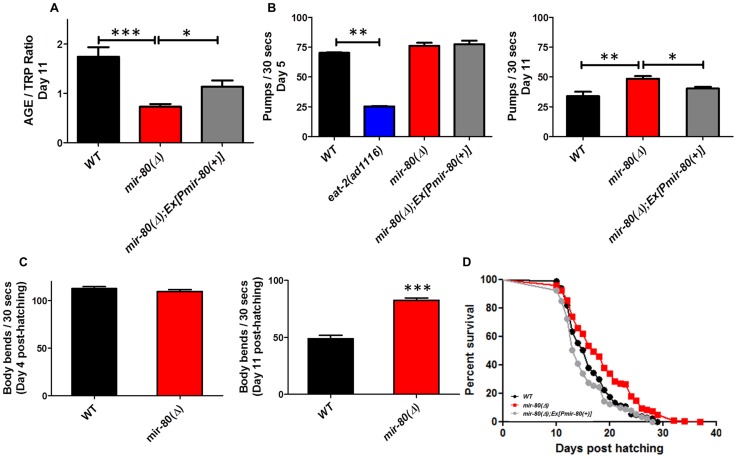
*mir-80*(Δ) exhibits multiple features of healthy aging. Fig. 1A. *mir-80*(Δ) has low intestinal age pigment levels compared to wild type during late adult life (day 11). We grew age-synchronized WT (black), *mir-80*(Δ) (red), and *mir-80*(Δ); Ex[P*mir-80(+)*] (grey) under standard conditions (20°C, on *E. coli* OP50-1) and scored animals for age pigment levels using a fluorimeter (n = 100 per strain/trial; day 11, as counted from the hatch; *mir-80*(Δ) is *nDf53; mir-80(+)* rescue transgene is *nEx1457* [18]). Age pigment fluorescence, which increases with age, is normalized to endogenous tryptophan fluorescence, which remains relatively constant with age [19], (AGE/TRP ratio ∼58% decreased in *mir-80(Δ)* vs. wild type). Graphs represent mean data from at least 3 independent trials. Data were compared using the One-way ANOVA followed by Newman-Keuls multiple comparison test, *** - p<0.0005, * - p<0.05; WT to Ex[P*mir-80(+)*] rescue p<0.12. In the rescued strain, age pigment levels might not reach WT levels due to mosaicism of the extrachromosomal transgene, the *mir-80* transgene dose, or “sponge” effects of overexpression. Fig. 1B. *mir-80*(Δ) maintains youthful pharyngeal pumping in late adulthood. We assayed age-synchronized WT (black), *mir-80*(Δ) (red), and *mir-80*(Δ); Ex[P*mir-80(+)*] (grey, *nEx1457* ([18])) for pharyngeal pumping rates on Day 5 (left) and Day 11 (right) (30 s interval, n = 10/trial, 3 trials). For day 5, we included the *eat-2(ad1116)* mutant (blue), impaired for pharyngeal pumping to ∼30% WT rate, as a negative control. In this assay we compared healthy appearing animals (most vigorous locomotion). Graph is of cumulative data from 3 independent trials. Data were compared using the One-way ANOVA followed by Newman-Keuls multiple comparison test. * - p<0.05; ** - p<0.005, *** - p<0.0005. *mir-80*(Δ) pumping rate is modestly higher than WT at day 5 (p = 0.023), but note that relative pumping differences at Day 5 are small compared to differences at Day 11 (∼44% increase). Fig. 1C. *mir-80*(Δ) maintains youthful swimming vigor in late adulthood. We assayed age-synchronized animals, WT (black), *mir-80*(Δ) (red), and *mir-80(Δ)*; Ex[P*mir-80(+)*] (grey) for swimming mobility at Day 5 and Day 11 post-hatching (n≥30, 3 independent trials are combined in presented data). Data were compared using 2-tailed Student's T-test, *** - p<0.0001. Although *mir-80*(Δ) and WT swim similarly in young adult life, *mir-80*(Δ) mutants better maintain swimming prowess late in life, ∼69% increased body bend rate. Fig. 1D. *mir-80*(Δ) mutants have increased mean and maximum lifespans. We assayed age-synchronized WT (black), *mir-80*(Δ) (red), and *mir-80*(Δ); Ex[*Pmir-80(+)*] (grey) animals grown under standard conditions (20°C, OP50-1) for viability (movement away from worm pick by gentle touch) at the indicated days. We initiated trials with relatively vigorous animals on day 9 from the hatch (10 animals per plate, ≥25 per strain per trial, 3 independent trials, which are combined here). Data from individual trials are shown in Fig. S1. Statistics were calculated using the Log-rank Test. *mir-80*(Δ) mutants exhibit a significant extension in lifespan as compared to WT (p<0.0001) and transgenic expression of *mir-80(+)* reversed the longevity increase (p<.0001).

Our previous studies revealed that age pigment levels (lipofuscin+advanced glycation end products) inversely correlate with locomotory healthspan—low age pigment levels late in life are typical of animals that age gracefully and maintain strong locomotory vigor, whereas high age pigment levels are typical of same-chronological age animals that age poorly and appear decrepit [19]. Thus, to identify *C. elegans* miRNAs that might impact healthy aging, we screened available *C. elegans mir* deletion strains [20] for differences in autofluorescent age pigment levels in old animals. We found that *mir-80(nDf53)* [hereafter referred to as *mir-80(Δ)*], exhibits low age pigment fluorescence levels late in life compared to wild type (WT) animals (Fig. 1A, ∼58% lower, p<0.0005). The low age pigment phenotype of *mir-80*(Δ) is rescued by a transgene array harboring a *mir-80(+)* gene, confirming that the low age pigment phenotype is conferred by *mir-80* deletion itself. Thus, late in adult life (∼2/3 through the WT lifespan), *mir-80*(Δ) mutants exhibit low age pigment accumulation typical of healthy aging animals.

To test if *mir-80*(Δ) mutants exhibited additional healthspan phenotypes, we next measured two indicators of maintained muscle integrity and function late into adult life–pharyngeal pumping rates and swimming vigor. Pharyngeal pumping is the mechanism by which food is pulled into the gut using specialized cardiac-like muscles. Pharyngeal pumping rates decline markedly with age, such that after the first week of life, feeding capacity is greatly diminished [21], a functional decline that tracks with physical changes in muscle integrity [22], [23], [24]. We find that pumping rates are significantly higher in *mir-80*(Δ) late in life (day 11) as compared to WT (44% increase), a phenotype reversed by a *mir-80(+)* transgene (Fig. 1B, right graph; p<0.005). Importantly, 5 day old WT and *mir-80*(Δ) (i.e., young adult; Fig. 1B, left graph) have similar pumping rates. Thus, *mir-80*(Δ) mutants are not simply hyper-activated for pumping, but rather maintain pumping function better late into life. We conclude that *mir-80*(Δ) exerts a positive effect on the quality of cardiac-like muscle aging.

As occurs with human skeletal muscle sarcopenia (the debilitating progressive loss of muscle mass and strength that accompanies aging across species), *C. elegans* body wall muscle deteriorates with age, featuring sarcomere loss [22], [24]. Physical decline is correlated with loss of locomotion vigor. We compared late-age swimming (body bend frequency) in WT and *mir-80*(Δ) to show that *mir-80*(Δ) mutants are significantly more vigorous swimmers in late adulthood (Fig. 1C right panel; 69% increase at day 11, p<0.0001). Early in adult life WT and *mir-80*(Δ) swim similarly (Fig. 1C, left panel). We conclude that *mir-80*(Δ) delays locomotory aging without altering young adult swimming behavior itself.

Given that *mir-80*(Δ) mutants exhibit several features of extended healthspan, we examined the longevity phenotype. We find that *mir-80*(Δ) mutants exhibit both mean and maximum healthspan extension, subject to *mir-80(+)* transgene rescue (Fig. 1D, p<0.0001, individual lifespan data in Fig. S1; average age increase at 75% mortality over all lifespan studies in this paper (13) was 24.1%+/−4.7%). Thus, deletion of *mir-80* confers longevity.

In summary, *mir-80*(Δ) confers multiple features of extended adult healthspan late in life: lowered intestinal age pigment accumulation, maintained pharyngeal pumping capacity, increased swimming vigor, and lifespan extension. Because *mir-80*(Δ) does not exhibit notable defects in development ([20], and our observations), it appears that *mir-80* has a predominant and focused impact on aging of the adult.

### *mir-80*(Δ) mutants appear dietary-restriction constitutive

#### Spectral properties of age pigments

**Figure 2:**
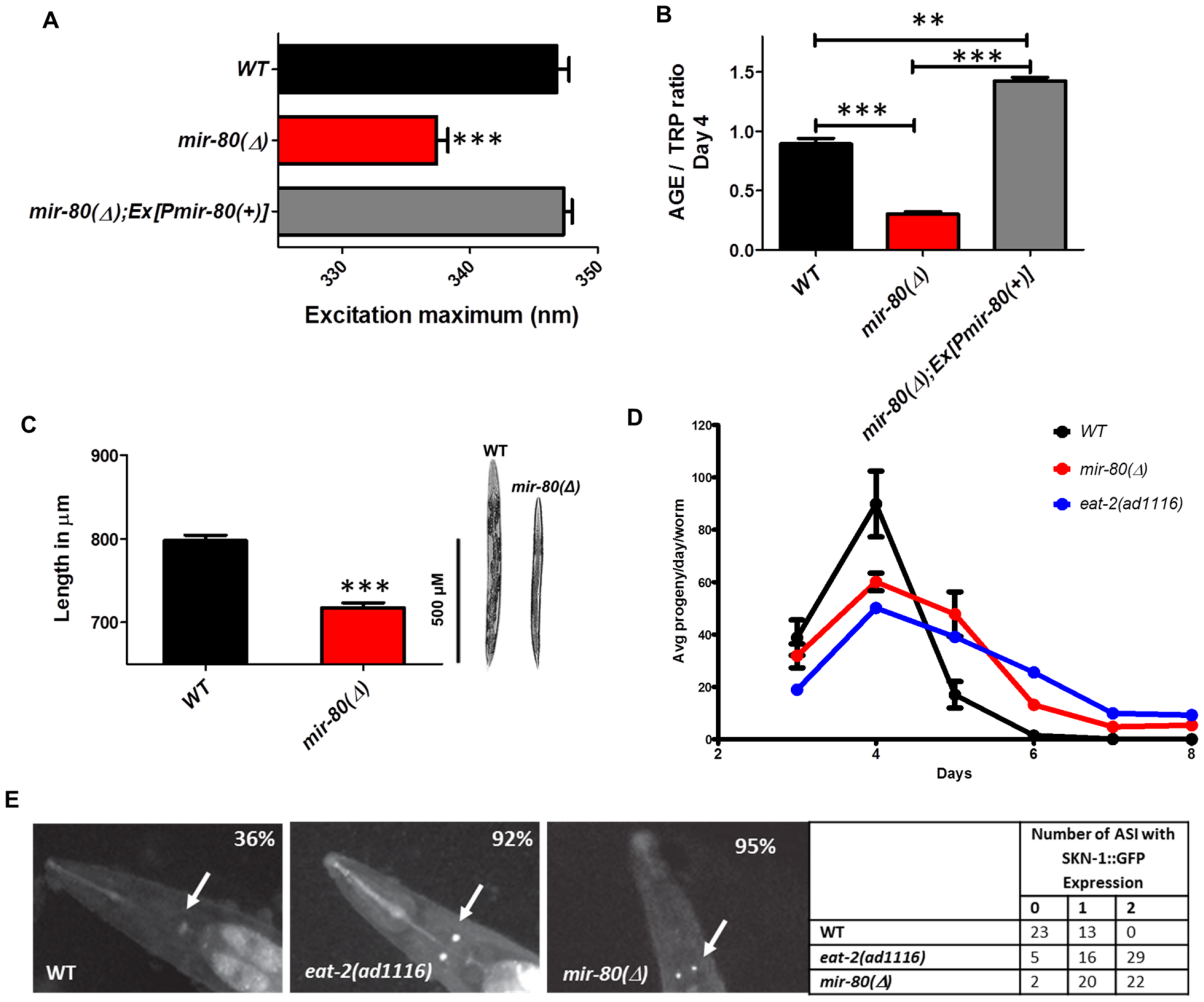
*mir-80*(Δ) exhibits multiple characteristics typical of DR animals. Fig. 2A. The *mir-80*(Δ) mutant exhibits the DR Ex_max_ shift. We assayed age-synchronized WT (black), *mir-80*(Δ) (red), and *mir-80*(Δ); Ex[P*mir-80(+)*] (grey) 4 day old animals grown under standard conditions (20°C, OP50-1). We used a spectrofluorimeter to scan transparent animals (n = 100 per strain/trial) to generate excitation/emission profiles as in [19]; wavelength of excitation at maximal fluorescence is indicated. Graphs represent mean data from at least 3 independent trials. Data were compared One-way ANOVA followed by Newman-Keuls multiple comparison test, *** - p<0.0005. *mir-80*(Δ) exhibits a significantly down-shifted Ex_max_ (p<0.0005) as compared to wild type under conditions of abundant food, a feature unique to DR [19]. Fig. 2B. The *mir-80*(Δ) mutant exhibits low age pigment levels early in life, as occurs in *C. elegans* DR. We assayed age-synchronized WT (black), *mir-80*(Δ) (red), and *mir-80*(Δ); Ex[P*mir-80(+)*] (grey) 4 day old animals grown under standard conditions (20°C, OP50-1). We scanned animals (n = 100 per strain) for fluorescence over a range of wavelengths, and normalized age pigment fluorescence (AGE) to tryptophan (TRP) fluorescence as in [19] for comparison. Graphs represent mean data from at least 3 independent trials. Data were compared using One-way ANOVA followed by Newman-Keuls multiple comparison test, *** - p<0.0005, ** - p<0.005. *mir-80*(Δ) exhibits low age pigment levels as compared to wild type (p<0.0005) early in life, which is true of all DR conditions previously tested (although not unique to DR). In these assays, levels were on average 66% lower in *mir-80*(Δ). Fig. 2C. *mir-80*(Δ) mutants are physically smaller than WT, typical of animals in DR. We measured age-synchronized WT (black) and *mir-80*(Δ) (red) 4 day old animals (examples at the right) grown under standard conditions (20°C, OP50-1) by imaging animals (WT n = 77, *mir-80*(Δ) n = 88) under DIC under low magnification. We measured using the segmented line tool in the ImageJ software by drawing a line across the length of the animal, and converted length in pixels to uM using a stage micrometer to assess image scale. We compared data using 2-tailed Student's T-test, *** - p<0.0005. *mir-80*(Δ) mutants are ∼10% shorter and look thinner than WT reared under the same conditions, typical of the scrawny appearance of animals in DR, example comparison on the right. Although size varies somewhat and is not as quantitative a measure as age pigment scores, we have used the scrawny appearance to identify likely *mir-80*(Δ) homozygotes in crosses. Fig. 2D. *mir-80*(Δ) mutants exhibit reduced fertility and an extended reproductive lifespan. We assayed egg production in age-synchronized WT (black), *mir-80*(Δ) (red), and DR mutant *eat-2(ad1116)* (blue) grown under standard conditions (20°C, OP50-1; parent n = 10, 3 independent trials). *eat-2* is one trial so bars are not provided. Data were compared using 2-tailed Student's T-test. Early in adult life, *mir-80*(Δ) produce a reduced number of live births per day (33% decrease, p<0.05 for Day 3; 180% increase, p<0.001 Day 4–6) and exhibit a prolonged reproductive lifespan (through Day 8 for *mir-80*(Δ) as compared to WT Day 6, p<0.001). The constitutive DR mutant, *eat-2* experiences a shift in reproductive lifespan (compared to WT, p<0.001) that is similar to *mir-80*(Δ). Fig. 2E. SKN-1-GFP, a molecular reporter of DR, is upregulated in *mir-80*(Δ) in the presence of food. SKN-1::GFP expression in the two ASI neurons is a molecular signal of some DR [7]. We constructed strains that included an integrated rescuing *skn-1-gfp* fusion gene expressed from the native *skn-1* promoter, *Is007[skn-1-gfp]* [7], and measured at Day 7, 20°C, growth in OP50-1 (white arrows). WT animals show low levels of ASI expression (36% with very weak expression in only one ASI), while DR-constitutive *eat-2(ad1116)* animals display constitutive expression of SKN-1-GFP in the ASI neurons (92% in 1 or 2 neurons, strong expression). 95% of *mir-80*(Δ) have 1–2 ASIs expressing at this timepoint. These data support that *mir-80*(Δ) mutants are in DR even when reared in the presence of ample food.

To ask whether *mir-80*(Δ) might act via a DR mechanism to extend healthspan and lifespan, we tested *mir-80*(Δ) mutants for phenotypic features of the DR state (Fig. 2). Our previous *in vivo* studies of fluorescent age pigments revealed that transparent *C. elegans* under DR have a unique fluorimetric “signature” that is distinct from spectral properties of both WT and long-lived mutants induced by other longevity pathways [19], [25], [26]. A spectrofluorimeter excitation/emission direct scan of WT reports a characteristic excitation maximum (Ex_max_) for age pigments at ∼345 nm. However, under all DR-inducing conditions we previously tested (multiple feeding-impaired mutants [27], liquid feeding of WT [7], [19], limiting bacterial concentrations for WT [4], complete removal of food [28], treatment with DR-mimetic drug metformin [25]), we noted a downward shift in Ex_max_. Thus, the age pigment Ex_max_ shift indicates a DR-like state. We found that *mir-80*(Δ) consistently exhibits the DR Ex_max_ shift despite growth in the presence of abundant food (Fig. 2A) and has low age pigment levels, even at young age (day 4, p<0.0005, Fig. 2B, about 66% lower in these studies), the latter of which also characteristic of DR mutants. Thus, *mir-80*(Δ) exhibits the spectral signature of DR despite the presence of food, consistent with *mir-80*(Δ) being a DR constitutive mutant.

#### Scrawny bodies

WT animals under DR appear thin and pale [29], [30]. We found that *mir-80*(Δ) had a somewhat scrawny and pale appearance and on average is ∼10% shorter in length than WT (p<0.0005, Fig. 2C). Thus, the physical appearance of *mir-80*(Δ) mutants resembles that of DR animals.

#### Reduced fecundity

Reduced fecundity is associated with DR across species [31]. We find that *mir-80*(Δ) exhibits a significant extension of reproductive period, producing progeny through Day 8 as compared to WT Day 6, p<0.001, Fig. 2D. In addition, there is a decrease in the number of live progeny laid per day by *mir-80*(Δ) (p<0.05 for Day 3 and p<0.001 for days 4–6), without a significant difference in the total number of surviving progeny (not shown), a pattern of progeny production similar to that of feeding-defective DR mutant *eat-2* (Fig. 2D). We conclude that *mir-80*(Δ) exhibits reduced fecundity, similar to animals experiencing DR.

#### Hypersensitivity to a DR-mimetic drug

The anti-diabetes DR-mimetic drug metformin can induce a life-prolonging DR-like state in WT animals, but administering metformin to animals already in DR (e.g., the *eat-2* mutant), leads to a reduced lifespan [25]. This metformin hypersensitivity in DR animals has been suggested to result from pushing DR metabolism into a deleterious starvation-like state [25], [32]. We found that although *mir-80*(Δ) is long-lived relative to WT under normal growth conditions (Fig. 1D), the *mir-80*(Δ) lifespan is decreased relative to WT in the presence of metformin (three individual trials and combined data in Fig. S2), similar to what occurs for DR mutant *eat-2*. Thus, like other DR strains, *mir-80*(Δ) is hypersensitive to metformin, consistent with *mir-80*(Δ) being in a DR constitutive state.

#### Molecular reporter of DR: SKN-1 expression in ASI neurons

SKN-1 is a transcription factor crucial for endodermal development and response to oxidative stress [33], that must also be expressed in the pair of chemosensory ASI neurons for the longevity outcomes of some DR regimens [7]. For example, SKN-1 appears continuously expressed in the ASI neurons in the constitutive DR *eat-2(ad1116);Is007[skn-1-gfp]* reporter strain, whereas this reporter is not highly expressed in wild type *Is007[skn-1-gfp]* animals that are grown under *ad lib* feeding conditions (Fig. 2E). We found that SKN-1-GFP is highly expressed in the ASI neurons in the well fed *mir-80*(Δ) mutant (Fig. 2E). At day 7, 95% *mir-80*(Δ) and 92% *eat-2* mutants exhibited strong signals in 1–2 ASI neurons; but 36% of WT only express weak signal in at best one ASI.

Our data support that *mir-80*(Δ) induces molecular features of DR.

In summary, the *mir-80*(Δ) mutant has a lean body, reduced fecundity, hypersensitivity to metformin, and expresses both molecular and fluorimetric markers of DR despite growth in abundant food. Importantly, unlike the *eat-2* mutant, pumping rates in *mir-80*(Δ) are normal in young animals and are actually enhanced relative to WT later in life (Fig. 1B). Thus, *mir-80*(Δ) does not physically reduce the ability to eat, but rather is likely to act further downstream to influence DR metabolism. Remarkably, then, deletion of a single miRNA gene can shift *C. elegans* metabolism into DR to promote healthy aging.

### *mir-80* expression is positively regulated by the presence of food

#### *mir-80* is broadly expressed in well-fed animals

**Figure 3:**
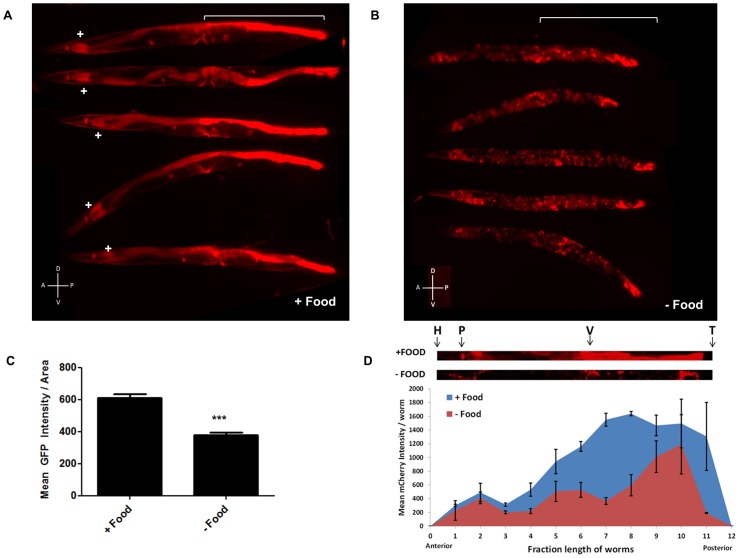
*mir-80* expression is generally high in the presence of food, but low when food is lacking. 3A. Examples of expression of extrachromosomal *bzEx207* [P*_mir-80L_*mCherry] line grown in the presence of unlimited *E. coli*. Note that this transgenic line, typical of 4 lines that have the long *mir-80* promoter region, exhibits substantial reporter expression in the first two cells of the intestine (indicated by white+sign) and in the posterior intestine (white bracket). Lower level expression is evident in several other tissues. Animals are adult day 6, but we find no bleed through of signals using red/green filter sets (Fig. S4B) so age pigments do not confound this analysis. 3B. Examples of expression of the *bzEx207* [P*_mir-80L_*mCherry] line grown in the presence of unlimited *E. coli* until young adulthood and then switched to no food for 48 hours. 6 day old adults are aligned with anterior to the left, posterior gut region indicated by white bracket. Most posterior gut fluorescence is markedly diminished, although expression in the anterior two intestinal cells, the central egg laying muscles, and the very posterior gut remains high. 3C. Quantitation of fluorescence signals for a *mir-80* promoter fusion reporter line in food vs. food limitation. Fluorescence of overall *bzEx207*[P*_mir-80L_*mCherry] line expression after 48 hrs on no-food plates. Food limitation in these studies was by dietary deprivation [28], but food dilution on solid NGM media [4] and food dilution in liquid media [4] induced similar changes in these lines (Fig. S4). Graph represents spectrofluorimeter measurements of fluorescence levels (whole body) for at least 50 animals per DR regimen. Pairwise comparisons were made using Two-tailed Students' T-test. *** - p<0.0005. Same exposure times were used for complementary panels. 3D. Analysis of food-regulated expression of p*_mir-80L_*mCherry expression along the nematode body implicates posterior intestinal regions as a major site of regulation. We compared p*_mir-80L_*mCherry signals in transgenic ZB3042 grown either in the presence of food (blue) or switched to no food for 24 hrs (red) (measured at day 4, n = 39). We used the ImageJ program to create a 25 pixel segmented line covering the animal and measured mean fluorescence intensity along the body, dividing the length into 12 equal bins and plotting the mean fluorescence intensity at each point. Representative animals are depicted above with the approximate body positions indicated (H = head, P = pharynx, V = vulva, T = tail). Note that although food regulation is apparent in most of the body, food-regulated expression changes in the regions of the mid- and posterior intestine are most dramatic. Error bars indicate standard error for each bin measurement.

Previous deep sequencing studies indicate that miR-80 is a relatively high abundance miRNA expressed from late embryogenesis into adulthood [34], [35]. Somewhat paradoxically, two published transgenic lines of the same P*_mir-80_*::GFP fusion transcriptional reporter (utilizing 1741 bp 5′ to *mir-80* as promoter [17]), exhibit different cellular expression patterns, an observation we confirmed (Fig. S3A,B). To address this discrepancy, we constructed a new mCherry reporter that extended *mir-80* 5′ sequences up to the next annotated gene (1814 5′ bp, designated P*_mir-80L_*). 5/5 extra-chromosomal transgenic lines of this reporter exhibit a broad cellular expression pattern, somewhat similar to the published extrachromosomal array line (vulva, hypodermis, body wall muscle, head neurons, tail neurons, excretory cell, dorsal/ventral nerve cord, and weaker expression in intestine [anterior or posterior] and pharynx). The P*_mir-80L_* pattern is distinctive in exhibiting highest expression in the two most anterior gut cells and in posterior gut under high food conditions (Fig. 3A). Interestingly, *mir-80* does not appear expressed in the ASI neurons in any lines (Fig. S4A,B) and thus miR-80 most likely acts non-cell autonomously to influence *skn-1*::GFP expression in the ASI neurons (Fig. 2E).

#### *P_mir-80_* reporter expression is down-regulated in the absence of food

Since the genetic elimination of *mir-80* results in constitutive DR phenotypes (Fig. 2, Fig. S2), we hypothesized that *mir-80* expression might be reduced when food is limited. We examined multiple *mir-80* transcriptional reporters for expression level 48 hours after shift from abundant food to no food (Fig. 3B–D, Fig. S3). We find that for all reporters examined, expression for *mir-80* is significantly lower in the absence of food. Furthermore, food limitation by alternative diet regimens is also associated with general down-regulation of *mir-80* reporter expression (Fig. S5). For the broadly expressing transgenes, it appeared that overall expression in many cells was down-regulated, although the changes in the mid and posterior intestine have the largest differential, ∼4–10× P*_mir-80L_*::mCherry level changes food/no food (Fig. 3D). We confirmed general down-regulation in the absence of food by deep sequence analysis of miR-80: overall expression levels food/no food are 1.5 increased (data not shown, p-value<0.05). However, we emphasize that not all cells exhibit miR-80 down-regulation: expression in two anterior-most and two posterior-most gut cells, and vulval muscle expression appear maintained, and possibly enhanced, in no food. We conclude that *mir-80* expression can be modulated by the presence of food: in most cells, *mir-80* expression is relatively high in the presence of food and is reduced when food is limiting. The broad *mir-80* expression pattern suggests a potential role for miR-80 in global regulation of metabolism; although dramatic posterior intestinal regulation raises the possibility that major changes in this tissue could provide the most critical influence on organism-wide regulation (see Discussion).

### Transcription factors implicated in DR metabolism are required for *mir-80(Δ)*-associated fluorimetric features

**Figure 4:**
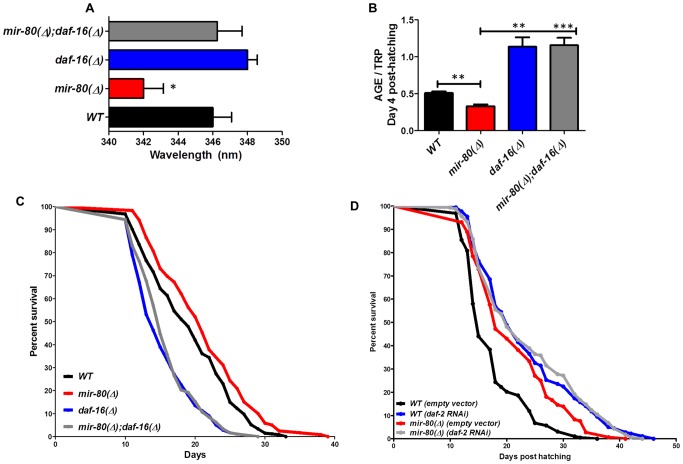
*daf-16*/FOXO is needed for the fluorimetric DR signature and longevity phenotypes of *mir-80*(Δ). Fig. 4A. Transcription factor *daf-16/FOXO* is required for the Ex_max_ shift phenotype in *mir-80(Δ)*. We reared age-synchronized animals under standard growth conditions (20°C, OP50-1) and measured age pigment spectral properties at Day 4 (50 animals per strain) for WT (black bar), *mir-80*(Δ) (red), *daf-16*(Δ) allele *mgDf50* (blue), and *mir-80*(Δ);*daf-16*(Δ) double mutant (grey). The same color coding is used for panels 4A–4D. We recorded Ex_max_ as the highest peak detected by the Datamax software package suite (Horiba Scientific). Graphs represent mean data from at least 3 independent trials. Data were compared using 2-tailed Student's T-test. *mir-80*(Δ) compared to WT * - p<0.05; *mir-80*(Δ);*daf-16*(Δ) double mutant compared to WT, ns. Deletion of *daf-16* reverses the Ex_max_ shift phenotype of *mir-80*(Δ). Fig. 4B. *daf-16*/FOXO is required for low age pigment levels in *mir-80*(Δ). We grew age-synchronized animals under standard conditions (20°C, OP50-1) and measured total age pigment fluorescence, normalized to total tryptophan fluorescence as in [19] (Day 4, 50 animals per trial). Graphs represent mean data from at least 3 independent trials. Error bars represent ±S.E.M. Data were compared using 2-tailed Student's T-test. *** - p<0.0005, ** - p<0.005. The low age pigment accumulation phenotype of *mir-80*(Δ) is reversed in the *mir-80*(Δ);*daf-16*(Δ) double mutant on day 4 (shown here) as well as on day 9 (data not shown). Fig. 4C. *daf-16* is required for the lifespan extension of *mir-80*(Δ). We grew age-synchronized animals under standard conditions (20°C, OP50-1). At day 9, we placed 10 healthy animals per plate, ≥40 per strain per trial, and we scored viability as movement away from pick touch on the indicated days. The graphs represent data combined from 3 independent trials. Statistics are calculated using the Log-rank Test. The *mir-80*(Δ);*daf-16*(Δ) double mutant is suppressed for the longevity phenotype of *mir-80*(Δ) (p<0.0001). We did not, however, observe dramatic overall changes in nuclear localization of DAF-16::GFP +/− *mir-80* (data not shown). Fig. 4D. *mir-80*(Δ) lifespan can be further extended by *daf-2(RNAi)*. We placed age-synchronized *mir-80*(Δ) L1 larvae (Day 1) on empty vector control (pL4440) or *daf-2* RNAi plates under standard conditions (20°C). At day 9, we placed 10 healthy animals per plate, ≥40 per strain per trial, and we scored viability as movement away from pick touch at the indicated days. The graphs represent data combined from 3 independent trials. Statistics are calculated using the Log-rank Test. *daf-2(RNAi)* increases the lifespan of *mir-80*(Δ) vector control (p<0.005), but additive effects for *mir-80*(Δ)*+daf-2(RNAi)* above the *daf-2(RNAi)* level are not observed (p = 0.98). Note that data from these experiments also provide a general sense of how *mir-80*(Δ) compares to *daf-2* for lifespan extension; roughly we find *mir-80*(Δ) effects are slightly less than half those of *daf-2(rf)*, see Table S4 for exact data from individual trials.

**Figure 5:**
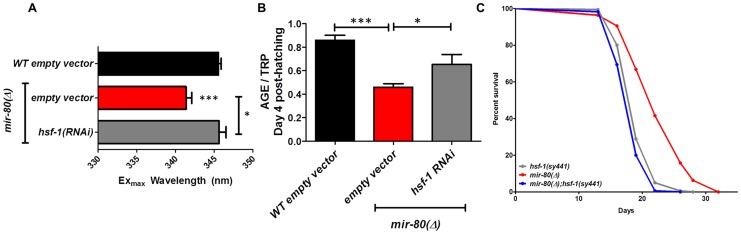
*hsf-1* is needed for the fluorimetric DR signature and longevity phenotypes of *mir-80*(Δ). Fig. 5A. *hsf-1(RNAi)* in the *mir-80*(Δ) background reverses the DR Ex_max_ shift. We grew age-synchronized animals under standard RNAi feeding conditions (20°C, HT115) and measured age pigments at Day 4 (50 animals per RNAi clone). We recorded Ex_max_ as the highest peak detected by the Datamax software package suite (Horiba Scientific). Black bar, WT+ empty vector RNAi; red bar, *mir-80*(Δ)+empty vector RNAI; grey bar, *mir-80*(Δ)*+hsf-1(RNAi)*. Graphs represent cumulative data from 3 independent trials. Error bars represent ±S.E.M. Data were compared using 2-tailed Student's T-test (** p<0.001). Note that *hsf-1(RNAi)* treatment of WT does not change Ex_max_ (data not shown). Fig. 5B. *hsf-1(RNAi)* in the *mir-80*(Δ) background partially counters the low age pigment level phenotype of *mir-80*(Δ). We grew age-synchronized animals under standard conditions (20°C, HT115) and measured total age pigment fluorescence, normalized to total tryptophan fluorescence as in [19] (Day 4 post-hatching, 50 animals per RNAi clone). Black bar, WT+ empty vector RNAi; red bar, *mir-80*(Δ)+empty vector RNAi; grey bar, *mir-80*(Δ)*+hsf-1(RNAi)*. Graphs represent cumulative data from 3 independent trials. Error bars represent ±S.E.M. Data were compared using 2-tailed Student's T-test (*** p<0.0001, * p<0.05 compared to *mir-80*(Δ) empty vector). Note that *hsf-1(RNAi)* treatment of WT does not change age pigment scores at day 4 (data not shown). Fig. 5C. *hsf-1* is required for *mir-80*(Δ)-induced longevity. We grew age-synchronized animals under standard conditions with low levels of FUDR to prevent progeny production (20°C, OP50-1, 50 uM FuDR). At day 9, we placed 10 healthy animals per plate, ≥40 per strain per trial, and we scored viability as movement away from pick touch at the indicated days. The graphs represent data combined from 3 independent trials. Statistics are calculated using the Log-rank Test. Error bars indicate ± S.E.M. The *mir-80*(Δ); *hsf-1(sy441)* double mutant is shorter lived than *mir-80*(Δ) (p<0.0001). Because RNAi knockdown is inefficient the nervous system (see [59]), the profound effects of *hsf-1(RNAi)* suggest that critical *hsf-1* and *mir-80* regulation occurs outside of the *C. elegans* nervous system.

**Figure 6:**
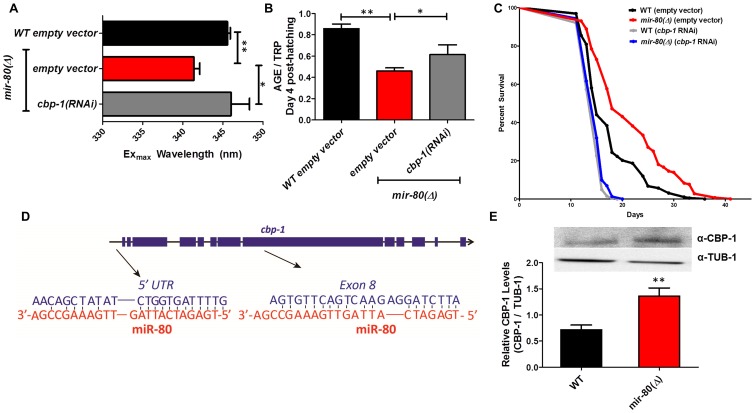
CBP-1 is critical for *mir-80*(Δ) healthspan benefits, and is a candidate direct binding target of miR-80. Fig. 6A. *cbp-1(RNAi)* in the *mir-80*(Δ) background reverses the DR Ex_max_ shift. We grew age-synchronized animals under standard RNAi feeding conditions (20°C, HT115) and measured age pigments at Day 4 (50 animals per RNAi clone). We recorded Ex_max_ as the highest peak detected by the Datamax software package suite (Horiba Scientific). Graphs represent cumulative data from 3 independent trials. Error bars represent ±S.E.M. Data were compared using 2-tailed Student's T-test (** p<0.001, * p≤0.055 compared to *mir-80*(Δ) empty vector). *cbp-1(RNAi)* Ex_max_ is comparable to that of *ad lib* wild type (p = 0.729). Note that *cbp-1(RNAi)* treatment of WT does not change Ex_max_ (data not shown), so this effect is specific to the DR signature of *mir-80*(Δ). Fig. 6B. *cbp-1(RNAi)* in the *mir-80*(Δ) background partially reverses low age pigment levels. We grew age-synchronized animals under standard conditions (20°C, HT115) and measured total age pigment fluorescence at day 4 (50 animals per RNAi clone), normalized to total tryptophan fluorescence as in ref. [19]. Graphs represent cumulative data from 3 independent trials. Error bars represent ±S.E.M. Data were compared using 2-tailed Student's T-test (** p<0.05, * p<0.1 compared to *mir-80(Δ)+*empty vector RNAi). Note that *cbp-1(RNAi)* treatment of WT induces modest reduction of age pigment levels (p = 0.01, data not shown). Fig. 6C. *mir-80*(Δ) longevity is dependent on *cbp-1*. We placed age-synchronized L1 larvae on empty vector control (pL4440) plates under standard conditions (20°C) until Day 4 (day 1 of adult life) at which time animals were moved to either empty vector control (L4440) or *cbp-1(RNAi)* plates. At day 9, we placed 10 healthy animals per plate (≥40 per strain per trial), and we scored viability as movement away from pick touch on the indicated days. The graphs represent data combined from 3 independent trials. Statistics are calculated using the Log-rank Test. *cbp-1(RNAi)* decreases the lifespan of *mir-80*(Δ) (p<0.0001 compared to vector control. Because RNAi knockdown is inefficient the nervous system (see [59]), the profound effects of *cbp-1(RNAi)* suggest that critical *cbp-1*/*mir-80* regulation occurs outside of the *C. elegans* nervous system. Fig. 6D. The *cbp-1* transcript includes two predicted binding sites for miR-80. Exon structure of *cbp-1* is indicated by thick blue bars, introns in thin black lines (see WormBase for details). The rna22 algorithm [10], which searches for target sites outside the 3′UTR, predicts that miR-80 binds *cbp-1* within the 5′ UTR and within exon 8. The potential alignments of miR-80 (red) to *C. elegans cbp-1* (blue) sequences are indicated. Note that the seed match to the exon 8 region is a perfect 10 bp match for *C. elegans*, and that the target sequence is conserved in mouse and human CBP1 (see Fig. S7). Fig. 6E. Endogenous CBP-1 protein levels are increased in 7 day old *mir-80(Δ)* mutants. We grew age-synchronized animals under standard conditions (20°C, OP50-1) and extracted total protein at Day 7 (100 animals per strain) for Western blot analysis (top). Graphs represent CBP-1 levels for each strain normalized to own TUB-1 levels. Error bars represent ±S.E.M. Data were compared using 2-tailed Student's T-test (** p<0.005). The graphs represent data combined from 3 independent trials. We noted that during young adulthood, native levels of CBP-1 seemed comparable to WT in *mir-80*(Δ), suggesting that additional regulatory controls are exerted on CBP-1 expression levels in development or early adulthood.

To identify genes required for *mir-80*(Δ)-regulated DR, we used RNAi to knockdown genes previously implicated in DR lifespan benefits, hypothesizing that genes required for *mir-80*(Δ) DR should be needed for the Ex_max_ shift and low age pigment levels typical of multiple DR states. Of the 18 genes we screened, we found that RNAi knockdown of transcription factors *daf-16*/FOXO, heat shock transcription factor *hsf-1*, and CREB binding protein homolog *cbp-1* modulated both the Ex_max_ shift and low age pigment levels of *mir-80*(Δ) (Tables S1, S2, Figs. 4A,B; 5A,B; 6A,B).

#### DAF-16/FOXO is required for fluorimetric indicators of DR age pigment and lifespan extension in *mir-80*(Δ)

Transcription factor *daf-16*/FOXO, an important modulator of longevity through insulin signaling, is also critical for lifespan extension benefits of serial dilution of bacteria on plates (sDR) and peptone dilution on plates (pDR) [4]. We found that the *mir-80*(Δ);*daf-16*(Δ) double mutant was reversed for both the DR Ex_max_ shift (Day 4, Fig. 4A, p<0.05) and the low age pigment levels (Fig. 4B, p<0.005) that are characteristic of *mir-80*(Δ). Moreover, the *mir-80*(Δ);*daf-16*(Δ) double mutant had a short lifespan, similar to that of *daf-16*(Δ) (Fig. 4C). These data identify DAF-16 as an required regulator of the fluorimetric DR signature and longevity benefits in the *mir-80*(Δ) background. Our *in silico* analyses did not identify candidate miR-80 target sites in the *daf-16* transcript, suggesting an indirect role in the *mir-80*(Δ)-regulated DR pathway.

To address the relationship of *mir-80*(Δ) and the insulin signaling pathway further, we compared longevity phenotypes of *mir-80*(Δ) and *mir-80*(Δ) treated with *daf-2* RNAi, which targets the *C. elegans* insulin receptor (Fig. 4D). We find that *mir-80(Δ)* lifespan can be further extended by *daf-2(RNAi*) (p<0.005). The additive effects of *mir-80*(Δ)*+daf-2(RNAi)* knockdown suggest that healthspan and longevity benefits of *mir-80*(Δ) may be conferred in part by a *daf-2*-independent pathway. However, the fact that *mir-80*(Δ) does not further extend *daf-2(RNAi)* lifespan (p<.98) is also consistent with a model in which miR-80 partially down-regulates the insulin pathway, and that *daf-2(RNAi)* reflects a stronger activation of the DAF-16-dependent transcriptional response, more toward an optimal healthspan signaling strength. Regardless of the details of pathway overlap, our data are definitive in establishing that *daf-16*/FOXO is needed for fluorimetric properties and longevity outcomes of *mir-80*(Δ).

#### hsf-1 deficiency eliminates multiple mir-80(Δ) healthspan phenotypes

hsf-1 regulates the expression of many heat-inducible target genes, modulates longevity, and is required for lifespan extension conferred by bacterial food deprivation [36] and dietary deprivation [5], [28]. In the mir-80(Δ) background, hsf-1(RNAi) reverses the Ex_max_ shift that typifies DR (p<0.06, Table S1, Fig. 5A) and partially restores 4 day age pigment levels (p<0.05 compared to mir-80(Δ)+empty vector RNAi, Table S2, Fig. 5B). hsf-1(RNAi) does not affect Ex_max_ or age pigment levels in WT (data not shown). To determine if hsf-1 is also required for mir-80(Δ) longevity, we examined survival curves for the mir-80(Δ);hsf-1(sy441) double mutant. We find that disruption of hsf-1 eliminates the lifespan extension conferred by mir-80(Δ) (Fig. 5C). We conclude that hsf-1 is required for both mir-80(Δ)-induced fluorimetric features that typify DR and for mir-80(Δ)-induced longevity. Consistent with a role for hsf-1 in mir-80(Δ)-induced benefits, HSF-1 target gene hsp-16.2 transcripts are elevated in the mir-80(Δ) mutant (Fig. S6). In silico analyses did not reveal candidate miR-80 target sites in the hsf-1 transcript, suggesting indirect regulation in the mir-80(Δ)-induced DR pathway.

#### The CREB-binding protein CBP-1 is required for mir-80(Δ)-dependent changes in DR fluorimetric indicators and for mir-80(Δ)-dependent longevity

C. elegans histone acetyltransferase transcriptional coactivator homolog cbp-1 is required for lifespan extension via at least three different DR regimens (growth in axenic media, growth in diluted bacteria in liquid media, and the eat-2 feeding-impaired model). In the bDR regimen, cbp-1 deficiency has been shown to disrupt expression of daf-16 and hsf-1 target genes [32], [37] and thus the action of two transcription factors that influence mir-80(Δ) benefits has been mechanistically linked to CBP-1 in DR. We find that cbp-1(RNAi) in the mir-80(Δ) mutant reverses the DR-associated Ex_max_ shift (p<0.05 +/− RNAi; Fig. 6A), and increases age pigment levels in day 4 animals (p<0.09, +/− RNAi, Fig. 6B) (cbp-1(RNAi) does not affect Ex_max_ but modestly reduces age pigment levels in WT (data not shown)). Thus, cbp-1 activity plays a role in mir-80(Δ) regulation of age pigments, and appears generally needed for mir-80(Δ) DR metabolism. Consistent with a contribution to DR benefits, we find that the lifespan extension conferred by mir-80(Δ) depends strongly on cbp-1 (p<0.003; Fig. 6C).

### Sequences within the *cbp-1* transcript may be direct binding targets of miR-80

Interestingly, of the three transcription factors required for *mir-80*(Δ) healthspan, *cbp-1* is the only one for which the transcript is predicted to include potential miR-80 miRNA target sequences (Fig. 6D). One candidate miR-80 binding site is present in the *cbp-1* 5′UTR, and another is present within exon 8. To test whether direct CBP-1 regulation might be a mechanism by which miR-80 controls metabolic state, we constructed translational reporters in which the *cbp-1* promoter drives expression of a GFP that includes either no candidate miR-80 binding sites (NBS) or both the 5′UTR and the exon 8 candidate binding sites (5+8BS) (Fig. S7A).

We compared GFP expression levels of these constructs in *ad lib* fed animals +/− *mir*-80, with a focus on the posterior gut region in which *mir-80* regulation is most dramatic. We find that the NBS construct is not regulated by *miR-80*(Δ) (Fig. S7B, left panel); whereas the 5+8BS construct is expressed at a higher level in the absence of *mir-80* (Fig. S7B, right panel). Although rigorous testing in native context will be required to validate *cpb-1* as a direct miR-80 target, our data suggest binding sites in the *cbp-1* transcript may contribute to *cbp-1* inhibition by miR-80 when levels are high in food.

If *mir-80* represses *cbp-1* translation, then we would expect higher levels of CBP-1 protein in *mir-80*(Δ) animals. We measured CBP-1 protein levels using anti-CBP antibodies against human CREBBP for WT and *mir-80*(Δ) mutants (day 7). We find that CBP-1 protein levels are significantly increased in *mir-80*(Δ) mutants compared to WT (p<0.05, Fig. 6E). Thus, in whole animal context, *mir-80*(Δ) is associated with increased CBP-1 protein.

**Figure 7:**
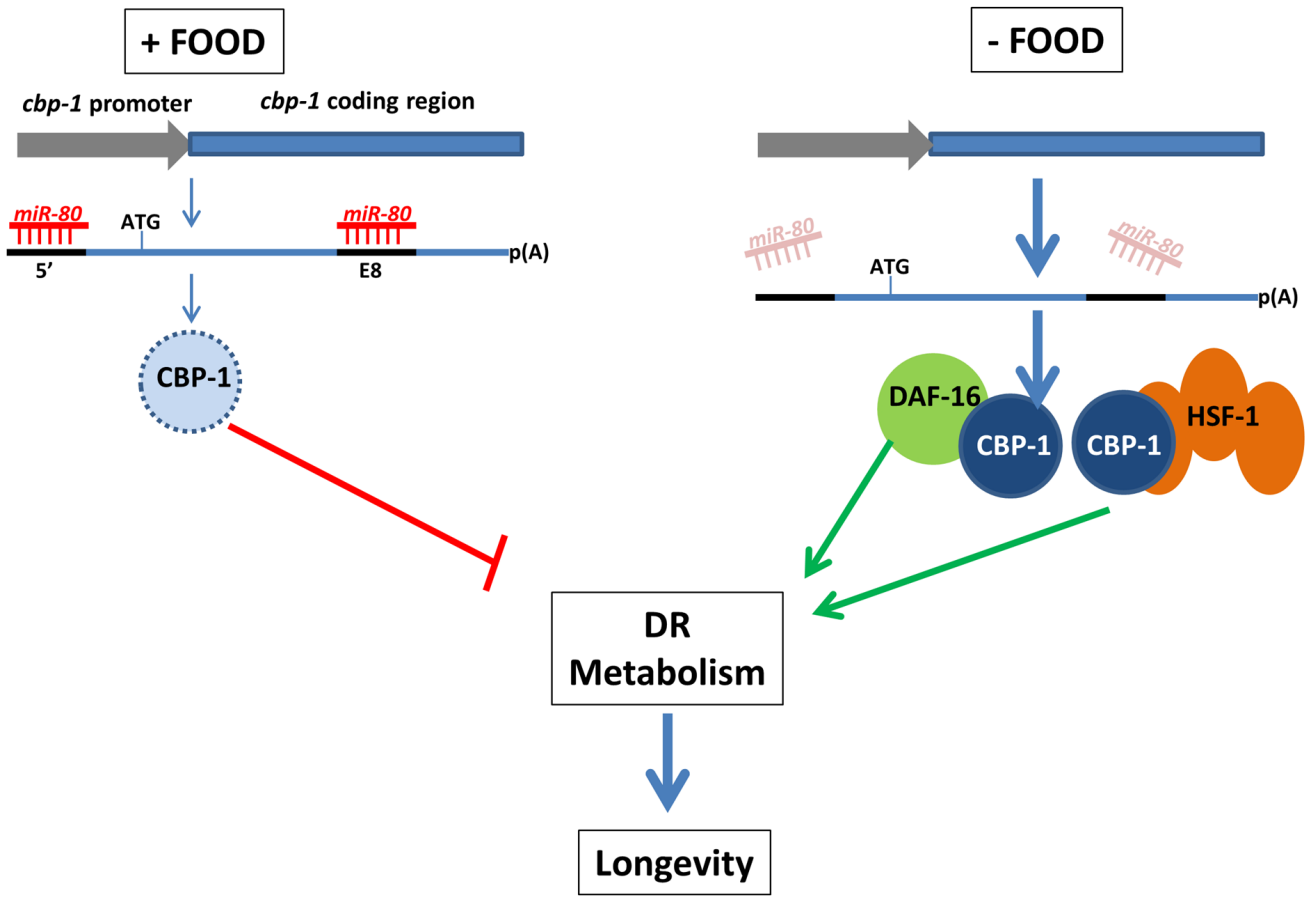
A model for miR-80 regulation of DR metabolism. In adults, when food is abundant, *mir-80* is expressed at a high level, and miR-80 binds to metabolic and signaling targets to down-regulate their expression. The *cbp-1* transcript, which includes two potential binding sites for miR-80, one in the 5′ UTR and one in exon 8 (exons thick dark blue lines, promoter lighter blue), and is essential for *mir-80*(Δ) benefits, is one candidate target (light blue represents relatively low CBP-1 concentration in food). When food is limiting, miR-80 levels drop, and translational repression of *cbp-1* could be relieved (dark blue circle represents higher concentration CBP-1). The CBP-1 protein associates with DAF-16 and HSF-1 to promote expression of genes required for DR metabolism and longevity. Note that although *cbp-1* is essential for *mir-80*(Δ) DR benefits, direct targeting remains to be proved and it is likely that additional targets help modulate the DR state. Since we cannot rule out that *daf-16*, *hsf-1*, and *cbp-1* disruptions make animals too generally sick to gain *mir-80*(Δ) benefits, alternative models are possible.

Our data are consistent with a model in which in the presence of food, *cbp-1* is translationally repressed by binding of miR-80 to target sites within the *cbp-1* transcript (Fig. 7). When food is lacking, miR-80 levels drop, translational repression of *cbp-1* is relieved, and CBP-1+DAF-16+HSF-1-mediated transcriptional changes induce DR within the animal. Interestingly, the human CREBBP transcript might be targeted by miR-80 family members or another miRNA homologous to the exon 8 site (Fig. S7C), suggesting miRNAs could exert a conserved role in DR metabolic regulation that might be harnessed in the future to promote healthy metabolism with anti-aging applications.

## Discussion

Deletion of a single *C. elegans* miRNA, *mir-80*, induces systemic healthy aging—improving cardiac muscle-like and skeletal-muscle-like maintenance and function later into life, limiting age-associated accumulation of lipofuscin-like material in the gut, and extending lifespan. Our data indicate that miR-80 acts as a negative regulator of metabolic loops that promote DR metabolism when nutrients are scarce. Acetyltransferase CBP-1 acts together with DAF-16/FOXO and HSF-1 to promote healthy metabolism in this regulatory circuit. Sequences within the Ce*cbp-1* transcript, the protein product of which increases in DR nematodes ([32], Fig. 6E) and in hypoglycemic mouse [38], may serve as direct targets of miR-80 down-regulation when food is abundant. Similarities between miR-80/target features in nematodes and mammals raise the possibility that miRNA manipulation of related DR metabolic loops in humans might be recruited to promote healthy aging.

### *mir-80* is expressed broadly and is regulated by food availability

*mir-80* is an abundant, widely-expressed miRNA, and thus might be involved in global regulation of metabolism coordinated across tissues. Indeed, multiple *mir-80* reporters indicate broad cellular expression and regulation by *E. coli* food availability. However, not all tissues/cells reflect similar magnitudes of regulation, with the largest fold food-induced change in expression in posterior intestine (Fig. 3D). The dramatic gut regulation raises the possibility that intestinal cells, well-positioned to monitor nutrient uptake, might play the most critical role in metabolic sensing and control. We speculate that miR-80 level changes in intestinal cells might initiate body-wide signaling via gut secretion of insulins and other hormones, analogous to human gastrointestinal tract and adipose tissue hormonal signaling to hypothalamus [39]. Because *mir-80*(Δ) induces *skn-1::GFP* expression in the ASI neurons (previously suggested to be similar to hypothalamic neurons [7]) but *mir-80* is not expressed in ASI neurons (Fig. S4A,B), relief of miR-80 repression under food limitation could act upstream of ASI *skn-1* induction via a gut-to-neuron signaling relationship.

### DAF-16, HSF-1 and CBP-1 transcription factors are needed for *mir-80*(Δ)-induced healthspan benefits in a likely complex regulatory circuit

The requirement for *daf-16*, *hsf-1*, and *cbp-1* in *mir-80*(Δ) DR is interesting in multiple regards. First, DAF-16/FOXO and HSF-1 can each individually bind to CBP-1 in nematodes and mammals (*C. elegans* DAF-16 and CBP-1; mammalian FOXO3A and CBP [40]; mammalian HSF-1 and CBP1 [41]), underscoring their capacity to co-regulate transcription. Second, previous work identified *C. elegans daf-16* and *hsf-1* as required for the CBP-1-dependent bDR lifespan extension [37]. In the bDR study, *cbp-1(RNAi)* blocked expression of DAF-16 and HSF target genes *sod-1* and *sip-1*, respectively, rather than blocking transcriptional induction of *daf-16* and *hsf-1* that accompanies bDR. These data suggest that the CBP-1 cofactor couples and modifies transcriptional outputs of DAF-16- and HSF-1-dependent longevity pathways under bDR conditions, a model likely to apply for *mir-80*(Δ)-induced DR.

Although our study focused on DR genes that have most dramatic impact on the age pigment DR signature, we emphasize that our data support that additional genes contribute in a complex network to regulate age pigment phenotypes in *mir-80*(Δ). For example, knockdown of either AMPK subunit encoded by the *C. elegans* genome, *aak-1* or *aak-2*, can alter age pigment levels (Tables S1 and S2), but not Exmax shift, suggesting separate regulation of lipofuscin content and levels. We thus anticipate that our data just touches the surface of a large interrelated network of metabolic genes and processes that are regulated by miR-80.

### Could miR-80 directly target the *cbp-1* transcript to regulate protein levels?

We fully expect that miR-80 regulates dietary restriction by binding to multiple target transcripts. An interesting candidate target, however, is the *cbp-1* gene itself, which we have shown to be critical for *mir-80*(Δ)-induced DR benefits. The potential *cbp-1* target sites for miR-80 binding are unusual, being situated in the 5′ UTR and within a highly conserved exon. Interestingly, the 5′ UTR sequences in *cbp-1* are perfectly conserved in *C. brenneri*, *C. briggsae*, and *C. remanei* (though not in *C. japonica*) and the exon 8 site is somewhat conserved among all (Fig. S7D). Exon targeting by miRNAs is common in plants [42] and has been demonstrated for mammalian transcription factors Nanog, Oct4, and Sox2 [43], [44], fly DICER [45], and is now predicted in many additional genes after algorithm refinements that consider coding sequences [45], [46].

Ideally, we could test direct miR-80 targeting *in vivo* by manipulation of a *cbp-1* transgene, +/− candidate miR-80 binding sites. Technical challenges, including the long length of the *cbp-1* gene/cDNA, as well as an apparent exquisite sensitivity of CBP-1 activity levels for health and viability [47], [48], precluded direct study. Our studies of expression of a GFP transgene flanked by the 5′ UTR and the exon 8 sites from *cbp-1* supported that miR-80 can down-regulate artificial construct expression in posterior gut. Although not definitive proof of direct targeting, these data, together with our findings that CBP-1 protein levels are elevated in DR (Fig. 6E; DR induction of CBP-1 also reported in [32], [37]) and miR-80 levels drop in DR (Fig. 3, Fig. S3, S5) are consistent with a model in which miR-80 mediates DR regulation by directly effecting CBP-1 levels (Fig. 7). Even if miR-80 effects are indirect, it is clear that *cbp-1* is critical for *mir-80*(Δ)-induced age pigment and lifespan changes. Given that *cbp-1* plays a role in dietary restriction associated with growth in axenic medium, growth on diluted bacteria, and *eat-2* feeding impairment [37] and intersects with the insulin pathway for lifespan extension [37], and that we have noted *mir-80* expression regulation under bacterial dilution and dietary deprivation, and a partial engagement of the insulin signaling pathway in *mir-80*(Δ)-induced longevity (Fig. 4D), the miR-80/CBP-1 regulatory loop may constitute a core mechanism by which diverse and intersecting metabolic pathways are coordinately regulated to respond to nutrient availability.

### Might the conserved miR-80 microRNA family regulate metabolism across species?

#### *C. elegans* miR-80 family

The most conserved *mir-80* family members encoded in the *C. elegans* genome are *mir-80*, *mir-58*, *mir-81* and *mir-82* [12], [18]. We did not find an Ex_max_ shift in *mir-58*(Δ) or in the double *mir-81*(Δ) *mir-82*(Δ) mutant (data not shown) and thus *mir-80* is the sole family member that can be deleted to induce the DR Ex_max_ shift. Interestingly, however, the quadruple mutant *mir-80; mir-58; mir-81-82* has a very small body size, more severe than the scrawny body type we documented for *mir-80*(Δ) (Fig. 2C), which can be rescued by a *mir-80* high copy number transgene [18] suggesting some functional redundancy among *mir-80* family members.

#### Drosophila melanogaster

*mir-80* is homologous to the miRNA bantam in *Drosophila melanogaster* (Fig. S7E), well studied for roles in developmental growth and cell death regulation [49], [50], [51], [52], and more recently implicated in regulation of the core circadian clock [53], neuronal dendritic growth regulation by epithelia [51], and regulation of ecdysone/insulin interplay that influences body size [54]. Our data raise the question of whether developmental processes or circadian clocks might be sensitive to metabolic state, or whether modulation of metabolism might be used to regulate growth.

#### Human

To date, there are 3 identified human miRNAs closely related to miR-80: hsa-mir-450b-3p; hsa-mir-556-5p, and hsa-mir-3689a-5p (Fig. S7E), none of which have been well studied for function in mammalian biology. The human hCBP coding sequences corresponding to Ce*cbp-1* exon 8 have sufficient homology to human miR-80 family members to raise the possibility of analogous interaction and conserved regulatory mechanism. We do note, however, that hCBP coding sequences in the Ce exon 8-homologus region have a stronger match to hsa-mir-136 (more distantly related to *Cemir-80)* (Fig. S7C). If the negative regulatory interaction between miR-80 (or specific miRNAs) and DR targets is conserved, disruption of human regulatory miRNAs might be exploited to promote healthy aging.

## Materials and Methods

We grew *C. elegans* under standard conditions [55] at 20°C, on *E. coli* strain OP50-1 or HT115 for RNAi. Age-synchronized cultures were prepared by bleach treatment egg preparation, with hatch counted as day 1. Note *all* presented data represent 3 independent combined trials; error bars +/− SEM. Protocol details are provided in Figure Legends and Supplemental Methods. Note *all* presented data in this paper represent at least 3 independent combined trials; error bars +/− SEM; we counted age with the egg hatch corresponding to day 0.

### Strains and plasmids

A detailed list of strains is included as Table S3.

We grew *C. elegans* under standard conditions [55] at 20°C unless otherwise indicated. The food sources we used were *E. coli* strain OP50-1 or HT115 for RNAi feeding experiments (*Caenorhabditis* Genetics Center, University of Minnesota, Twin Cities, MN, USA). To generate synchronized cultures, we bleached gravid adults and starved L1 progeny. The wild-type strain was var. Bristol N2 [55]. The *mir-80(nDf53)* allele breakpoints are 5′- tgctttcgatgtctatactctc -3′ and 5′-tctggcgaacgaaatgagt-3′, encompassing part of the promoter region, the entire precursor sequence and ∼300 bp downstream. We genotyped *mir-80(Δ)* by PCR using primer pairs mir80Out-F (5′- ttcgtcgccatcaacacacg-3′)+mir80Out-R (5′- gagcgcggatagatatacagtcag-3′) that flank the deletion and mir80Flank-F (5′- caacaacgatgtgaatgctcgtc-3′)+mir80Flank-R (5′- ctcgcacacggacggactgcc-3′) that bind internal to *nDf53*. We worked with a 6× outcrossed line. The *mir-80* deletion mutant does not exhibit gross developmental phenotypes ([20]; our observations). Developmental timing, L1 nuclei numbers, early adult locomotion, pumping rates, defecation rates, amphid neuron dye filling, and dauer entry/exit behaviors are within wild type ranges in *mir-80*(Δ), supporting that *mir-80* does not contribute an essential role in development and basic function. Thus, *mir-80* deletion primarily impacts adult maintenance and DR phenotypes.

For the P*_mir-80L_*:mCherry transcriptional reporter, we amplified the *mir-80* promoter using primers 5′-cgagatgagaagtaagaagagtgg-3′ and 5′-tccgtgtgcgagagagtgagcgag-3′ and cloned into the Pmec4::mCherry plasmid vector at the start codon of mCherry from [56] using the In-fusion cloning kit (Clontech Inc). The resulting plasmid was injected at 50 ng/ul into wild type animals along with a *rol-6* co-injection marker (100 ng/ul) to generate extrachromosomal transgenic lines ZB3039-ZB3043.

1. For the binding site test constructs (Fig. S7A), we amplified the *cbp-1* promoter (4.4 kb until start codon) using primers 5′-gACTAGTc tcttcc atgtcg gtttaa gcgcgg aaacgg tttttt aaa-3′ and 5′-tcccCCCGGGggga caatta gtagaa aaatgt atatat ttgac-3′ containing Spe1 and Xma1 restriction sites, respectively. This product was introduced within the Spe1-Xma1 digested pKS(-) vector. The *mir-80* target sites were incorporated within primers that amplified the GFP coding sequence using the primer sites (outlined below) and introduced at the Xma1 site of the above cloned P*_cbp-1_* vector: NBS: 5′- tccccccgggatgagtaaaggagaagaacttttcactgg-3′+5′-cggggtaccctatagttcatccatgccatgtgtaatccc-3′; 5+8 BS: 5′- tccccccgggaacagctatatctggtgatttgatgagtaaagaagaag-3′+5′-cggggtacctaagatcctcttgactgaacacttcatagttcatccatgcc-3′

### Measurement of fluorescent age pigments

We grew age-synchronized animals (see above) under standard conditions (20°C, OP50-1) and scanned animals (n≥50 per strain) for age pigment accumulation (Day 4, Day 9, Day 11) using a Fluorolog 3 spectroflorimeter as in Gerstbrien *et al.* [19]. All graphs represent mean data from at least 3 independent trials. For Ex_max_ determination at Day 4, we used Datamax software (Horiba Scientific) to identify the peak excitation value. The peak for tryptophan fluorescence was also analyzed to normalize scores, as TRP levels do not change markedly with age.

### qRT-PCR experiments for assaying gene expression changes

We synchronized strains by alkaline bleaching [57] and placed synchronized L1 larvae (Day 1) on NGM plates seeded with OP50-1 bacteria. On Day 4 or day 7, we moved approximately half the animals to plates containing OP50-1 with 50 uM FUdR. We used the other half for total RNA extraction using TRIZOL as described below. ∼1.5 ug of total RNA was used for cDNA synthesis using the Invitrogen SuperScript III cDNA synthesis kit and OligoDT primers to synthesize cDNA from all poly-adenylated RNA. We used 100 ng of cDNA to measure gene expression levels using the standard curve approach. Standard curves were generated from wild type cDNA by utilizing multiple dilutions of cDNA (1000, 100, 10, 1, 0.1, 0.01 ng) and probing for expression levels of the house-keeping gene, actin (*act-1*). Primers used were act1RT-F (5′- ttactctttcaccaccaccgctga-3′) and act1RT-R (5′- tcgtttccgacggtgatgacttgt -3′) for *act-1*, ama1RT-F (5′- cctacgatgtatcgaggcaaa-3′) and ama1RT-F (5′- cctccctccggtgtaataatg-3′) for *ama-1*, hsf1RT-F (5′-tagtaatggcagagatgcgtgcga-3′) and hsf1RT-R (5′- tggctgcatgacagagacgagaaa-3′) for *hsf-1* and hsp16.2RT-F (5′- atggaacgccaatttgctccagtc-3′) and hsp16.2RT-R (5′- tccttggattgatagcgtacgacc-3′) for *hsp-16.2*. We plotted C_t_ values obtained from amplification for target DR genes against this standard curve to determine transcript levels.

### Quantification of GFP fluorescence from *mir-80* target site constructs

We synchronized strains (refer Fig. S7A,B, and Table S3) by alkaline bleaching [57] and placed synchronized L1 larvae (Day 1) on NGM plates seeded with OP50-1 bacteria. On Day 4, 100 mCherry(+) animals were picked and GFP fluorescence was measured in the spectrofluorimeter at 488 nm excitation and 511 nm emission. We measured fluorescence using ImageJ with a region-of-interest (ROI) that included the entire length of the body (using the line tool) and then plotted a histogram of the mean intensity along the length of the line.

### Western blot analysis for detection of CBP-1 protein

We synchronized strains by alkaline bleaching [57] and placed synchronized L1 larvae (Day 1) on NGM plates seeded with OP50-1 bacteria. On Day 7, 250 animals were placed in 50 ul of RIPA buffer (20 mM Tris-HCl pH 7.5, 150 mM NaCl, 1 mM Na_2_EDTA, 1 mM EGTA, 1% NP-40, 1% Sodium deoxycholate, 2.5 mM beta-glycerophosphate, 1 mM Na_3_VO_4_)+Protein sample buffer and heated at 95°C for 15 mins. 25 ul of samples was loaded onto a MiniProtean TGX gradient gel (4–20%, Bio-Rad) and transferred onto PVDF membrane following separation. Membrane was blocked using 5% non-fat milk in PBST buffer for 1 hour. Membrane was then incubated with CBP-1 and TUB-1 antibodies (Santa Cruz Biotechnology) at 1∶500 and 1∶4000 dilutions respectively in 2% non-fat milk overnight at 4°C. Protein bands were detected using the ECL reagent (Invitrogen) using horseradish peroxidase conjugated secondary antibodies (Jackson ImmunoResearch Labs) at 1∶10,000 dilutions. Band intensities were calculated using ImageJ [58].

## Supporting Information

Figure S1 Individual lifespan data for lifespan analysis of *mir-80(Δ)*. We grew age-synchronized WT (black), *mir-80(Δ)* (red) or the *mir-80(+)* (grey) under standard plate conditions (200C, OP50-1). At day 9, we placed 10 healthy animals per plate, and we scored viability as movement away from pick touch on the indicated days. Statistics were calculated using the OASIS software. Details are presented in Table format. Three additional trials that did not include the rescued strain, as well as other trials featured in the text, showed similar trends, on the order of 10–30% lifespan extension. Note the some trials with *mir-80* transgene rescue suggest that overexpression of *mir-80* may be deleterious, and that in a small minority of trials, we did not see life extension although the culture always trended in that direction.
(TIF)

Figure S2 The *mir-80*(Δ) mutant exhibits hypersensitivity to the DR-mimetic drug metformin, similar to DR mutant *eat-2*. We grew age-synchronized WT (black), *mir-80*(Δ) (red) or the *eat-2* mutant (blue) under standard plate conditions supplemented with 50 mM metformin (20°C, OP50-1). At day 9, we placed 10 healthy animals per plate, ≥40 per strain per trial, and we scored viability as movement away from pick touch on the indicated days. The upper left graph (A) represents data combined from 3 independent trials, which are presented individually in the other panels (B-D). Statistics are calculated using the Log-rank Test. Error bars indicate ± S.E.M. Metformin reduces lifespan for *mir-80*(Δ) and *eat-2* as compared to WT.
(TIF)

Figure S3 Published *mir-80::GFP* reporters are regulated by food availability. Fig. S3A. Example of expression in the Ex[P*_mir-80::_*GFP] line VL211 ([17], *wwEx18*) grown in the presence of unlimited *E. coli*. Animals are at day 6 from the hatch for all images in this figure. + indicates anterior. Fig. S3B. Excretory duct cell in the Is[P*_mir-80::_*GFP] in line VT1492 ([17], *maIs196*) grown in the presence of unlimited *E. coli*. White arrow indicates the fluorescent cell that is well labeled in this integrated line, tentatively identified as the excretory duct cell. . Example of expression of Ex[P*_mir-80_^::^*GFP] in line VL211 grown in the presence of unlimited *E. coli* to young adulthood and then switched to no food for 48 hours. Fig. S3D. Example of Is[P*_mir-80_^::^*GFP] line VT1492 excretory duct cell (white arrow) grown in the presence of unlimited *E. coli* to young adulthood and then switched to no food for 48 hours. Fig. S3 E,F. Quantitation of fluorescence signals for *mir-80* promoter fusion reporter lines in food vs. food limitation. (E) Fluorescence of overall Ex[P*_mir-80_^::^*GFP] line VL211, (F) excretory duct cell Is[P*_mir-80_^::^*GFP] line VT1492; after 48 hrs on no-food plates. Food limitation in these studies was by dietary deprivation [5], but food dilution on solid NGM media [8] and food dilution in liquid media [37] induced similar changes in these lines (see Fig. S5). Graphs represent measured fluorescence levels (whole body for E; cell region for F) for at least 50 animals per DR regimen. Error bars represent mean intensity ± S.E.M. Pairwise comparisons were made using Two-tailed Students' T-test. *** - p<0.0005, ** - p<0.005. Same exposure times were used for complementary panels.
(TIF)

Figure S4 A,B. Two different transcriptional reporters for *mir-80* fail to co-localize with ASI sensory neurons, so up-regulation of *skn-1*::GFP expression in those neurons in DR may be a non-autonomous consequence of miR-80 activity. The expression of transcription factor *skn-1* in the two head ASI sensory neurons can be necessary for DR lifespan extension benefits [7], and we have shown that *mir-80*(Δ) increases the expression of a *skn-1*::GFP reporter in the ASI neurons (Fig. 2E). Thus, an important mechanistic question is whether miR-80 is present in the ASI neurons where it might cell-autonomously affect *skn-1* expression. To test for *mir-80* expression in ASI neurons, we took advantage of the fact that ASI neuron endings are open to the environment and can uptake fluorescent dye from their surroundings. We used a dye-filling assay to label the amphid sensory neurons in the P*_mir-80_* fluorescent reporter lines to test for co-expression (P*_mir-80::_*GFP with red DiO, and P*_mir-80L::_*mCherry with yellow DiI). We reared animals under standard conditions (ad lib OP50-1, 20°C). We labelled {A} VL211 expressing P*_mir-80::_*GFP with red DiO, and reciprocally, {B} strain ZB3042 containing *bzEx207*[P*_mir-80L::_*mCherry] with yellow fluorescent DiI, using a standard protocol that enables the amphid neurons that are open to the environment (ASI, ADL, ASK, AWB, ASH and, ASJ) to dye-fill. For both approaches, we never observed co-label of the *mir-80* reporter with any amphid neurons (n = 30 per reporter); white arrows indicate ASI in representative images. Thus, although *mir-80*(Δ) influences *skn-1*::GFP expression in ASI neurons in older animals (Fig. 2E), expression data suggest miR-80 does not act cell autonomously in ASI neurons to exert this regulation. C. Absence of fluorescent bleed-through in through the GFP/DAPI filter sets in the P*_mir-80L_*::mCherry lines P*_mec-4_*::GFP (top row) and P*_mir-80_*::mCherry (bottom row) lines were age-synchronized via alkaline bleaching and plated on standard NGM containing OP50-1 as food. Day 4 animals were imaged using 3 filter sets – DIC (left), FITC (to image GFP, middle) and Texas Red (to image mCherry, right). Touch neurons labeled with GFP in the P*_mec-4_*::GFP strain are clearly and distinctly visible using the FITC channel (white arrows) and are absent in the Texas Red channel. Conversely, mCherry signal from a transcriptional reporter of mir-80 is clearly visible in the TexasRed channel (white arrows) but is absent in the FITC channel. Thus, our images of mCherry in adult intestine is unlikely to inadvertently score age pigment fluorescence.
(TIF)

Figure S5 *mir-80* is regulated by food availability under multiple food restriction conditions and as assayed with multiple reporters. We grew strains either under abundant food conditions or using the dietary limitation protocol indicated. Graphs represent measured fluorescence levels from areas indicated for 2 trials of at least 50 animals per DR regimen (except liquid DR for VL211, 17 worms as the strain bagged frequently), measured 48 hours after dietary limitation. Error bars represent Mean Intensity ± S.E.M. Pairwise comparisons were made using Two-tailed Students' T-test, ** - p<0.0005, ** - p<0.005. a) overall p*_mir-80_*GFP line VL211 expression, food dilution on solid NGM media. b) overall p*_mir-80_*GFP line VL211 expression, food dilution in liquid media. c) excretory duct cell p*_mir-80_*GFP line VT1492 expression, food dilution on solid NGM media. d) excretory duct cell p*_mir-80_*GFP line VT1492 expression, food dilution in liquid media. e) overall fluorescence p*_mir-80L_*mCherry expression for 4 independently derived lines, in abundant food or under dietary deprivation. In individual trials, 4/5 tested lines exhibited significant differences; one line was not regulated in this direction (not shown).
(TIF)

Figure S6 Relative HSF-1 target gene *hsp-16.2* transcripts are elevated in *mir-80(Δ)*, but HSF-1 transcript levels are maintained. Temperature-induced HSF-1 activation occurs post-translationally, via formation of an active trimer from inactive monomers that persist under basal conditions [60]. To address the question of whether *mir-80*(Δ) mutants exhibit increased HSF-1 transcriptional activity compared to WT, we used qRT-PCR to measure transcript levels of HSF-1 target gene *hsp-16.2* [61], +/− *mir-80*. Our data suggest that loss of *mir-80* indirectly upregulates HSF-1 activity to increase expression of HSF-1-dependent target genes. A. Transcriptional expression of HSF-1 target gene *hsp-16.2* is elevated in *mir-80*(Δ). We grew age-synchronized WT and *mir-80*(Δ) under standard conditions of abundant food (20°C, OP50-1) and harvested animals at Day 4 for total RNA isolation. We normalized raw qPCR scores for *hsp-16.2* (an *hsf-1* target) to *ama-1*. Graphs represent cumulative data from 3 independent trials with 3 technical replicates per trial. Error bars represent ±S.E.M. for RAW transcript gene values normalized to RAW *ama-1* levels. Normalized transcript levels of *hsp-16.2* are elevated in *mir-80*(Δ), * p<0.1 (2-tailed Student's T-test). B. *hsf-1* transcript levels (day 4) are not changed by the absence of miR-80. We grew age-synchronized WT animals in abundant food and harvested animals at Day 4 for RNA isolation. Raw qPCR scores for *hsf-1* were normalized to *ama-1*. Graphs represent cumulative data from 3 independent trials with 3 technical replicates per trial. Error bars represent ±S.E.M for RAW transcript gene values normalized to RAW *ama-1* levels. In Day 4 animals normalized *hsf-1* transcript levels were similar in WT and *mir-80*(Δ) (p = 0.90; 2-tailed Student's T-test). We noted a trend toward lower *hsf-1* transcript levels at day 7 (not shown), which we think reflects modulation of the dynamic transcriptional network altered by miR-80. C. Transcriptional expression of HSF-1 target gene *hsp-16.2* is elevated in *mir-80*(Δ). We grew age-synchronized WT (black bars) and *mir-80(Δ)* (red bars) under standard conditions of abundant food (20°C, OP50-1) and harvested animals at Day 4 for total RNA isolation. We normalized RAW qPCR scores for *hsp-16.2* to *act-1*. Graphs represent cumulative data from 3 independent trials with 3 technical replicates per trial. Error bars represent ±S.E.M for RAW transcript gene values normalized to RAW *act-1* levels. Data were compared using 2-tailed Student's T-test. Normalized transcript levels of *hsp-16.2* are elevated in *mir-80*(Δ), * p<0.07. D. *hsf-1* transcript levels are not regulated by the presence of miR-80 at day 4. We grew age-synchronized WT (black bars) and *mir-80*(Δ) (red bars) under standard conditions of abundant food (20°C, OP50-1) and harvested animals at Day 4 for RNA isolation. RAW qPCR scores for *hsf-1* were normalized to *act-1*. Graphs represent cumulative data from 3 independent trials with 3 technical replicates per trial. Error bars represent ±S.E.M. for RAW transcript gene values normalized to RAW *act-1* levels. Data were compared using 2-tailed Student's T-test. In Day 4 animals normalized *hsf-1* transcript levels were similar in WT and *mir-80*(Δ) (p = 0.14 *act-1*). We noted a trend toward lower *hsf-1* transcript levels at day 7 (not shown) that we think reflects modulation of the dynamic transcriptional network altered by miR-80.
(TIF)

Figure S7 miR-80 may directly target *cbp-1* mRNA in the posterior gut, is a conserved miRNA and potential binding sites for miRNAs in human CREBBP are present at sites analogous to those in *C. elegans*. A. Constructs used to test candidate miR-80 binding sites present in *cbp-1* for roles in translational repression. We used the native *cbp-1* promoter to express GFP reporters that included i) GFP lacking any candidate miR-80 binding sites (NBS); ii) the 5′ UTR candidate miR-80 binding site and the exon 8 candidate miR-80 binding site from *cbp-1* (5+8BS). B. Shown are GFP intensities for extrachromosomal p *_cbp-1_*GFP constructs without candidate miR-80 binding sites (NBS, left panel) or with the 5′ and exon 8 candidate miR-80 binding sites added (5+8BS); in WT (black line) or *mir-80*(Δ) (red line) backgrounds, day 7, n>30, posterior gut. Animal segments imaged and fluorescence measured as in Fig. 3. The 5+8bs construct is expressed at a higher level when *mir-80* is lacking, whereas the NBS construct is not. The 5+8BS high copy number construct also variably exhibited some “sponge” effects that might be attributed to titrating out endogenous miR-80 and family members (not shown). C. Exon structure of *C. elegans cbp-1* is indicated by thick blue bars, introns in thin blue lines (see WormBase for details). The rna22 algorithm [62] predicts that miR-80 binds *cbp-1* within the 5′ UTR and within exon 8. The potential alignments of miR-80 (red) to *C. elegans cbp-1* (blue) sequences are indicated. Note that the seed match to the exon 8 region is a perfect 10 bp match for *C. elegans*, and that the target sequence is conserved to some degree in mouse and human CBP1. However, human miR-136 is even a better match in this region (shown here). D. The predicted miR-80 target sites are conserved in the various *Caenorhabditis* spp. E. Alignments of miR-80 family members from *Drosophila melanogaster* (*bantam*) and human with *C. elegans* miR-80. Seed region is indicated by black bar; yellow highlight, full conservation; blue highlight, conserved. Note that there are 4 close members of the miR-80 family encoded in the *C. elegans* genome (*mir-58, mir-81, mir-82*; see [12], 2008, for alignments and discussion).
(TIF)

Table S1 An RNAi screen identifies *hsf-1* and *cbp-1* as required for the DR-associated Ex_max_ shift phenotype in the *mir-80*(Δ) background. We examined the literature for genes experimentally implicated in DR and used RNAi to knock these genes down in the *mir-80*(Δ) background (Day 4, 20°C, three independent trials, 50 animals/trial). Since the Ex_max_ shift is diagnostic of the DR state, we reasoned that genetic interventions that reversed the shift would define genes needed for the *mir-80*(Δ) DR pathway. P-values were calculated using two-tailed unpaired Students T-test; gene knockdowns that confer statistically significant p-values are in bold.
(PDF)

Table S2 RNAi directed against known DR genes in the *mir-80*(Δ) background identify genes required for the low age pigment level phenotype. We used RNAi to knockdown genes previously implicated in DR in the *mir-80*(Δ) background (Day 4, 20°C, three independent trials, 50 animals/trial) and quantitated age pigment levels relative to endogenous Trp levels. Since low age pigment/Trp ratios (AGE/TRP) are associated with the DR state [19], we reasoned that genetic interventions that elevated age pigment levels would help identify genes needed for the *mir-80*(Δ) DR pathway and/or for enhanced healthspan. P-values were calculated using the Unpaired two-tailed Student's T-test, values within the 90% confidence level are bold, increase or decrease indicated by arrows.
(PDF)

Table S3 Strains used in this study.
(PDF)

Table S4 Summary of lifespan experiments. Median lifespan is calculated from the GraphPad Prism Software while Maximum lifespan was calculated by the mean lifespan of the oldest 10% cohort in each experiment. P-values are calculated from Log-Rank Tests for the entire population for the complete lifespan.
(PDF)

Text S1 Supplementary methods.
(PDF)

Text S2 References used exclusively in the supplemental information.
(PDF)
