# Supplementary figures and images for: Deletion of microRNA-80 Activates Dietary Restriction to Extend C. elegans Healthspan and Lifespan
Source: PLoS Genet. 2013 Aug 29;9(8):e1003737. doi: 10.1371/journal.pgen.1003737 (PMC3757059; doi:10.1371/journal.pgen.1003737)

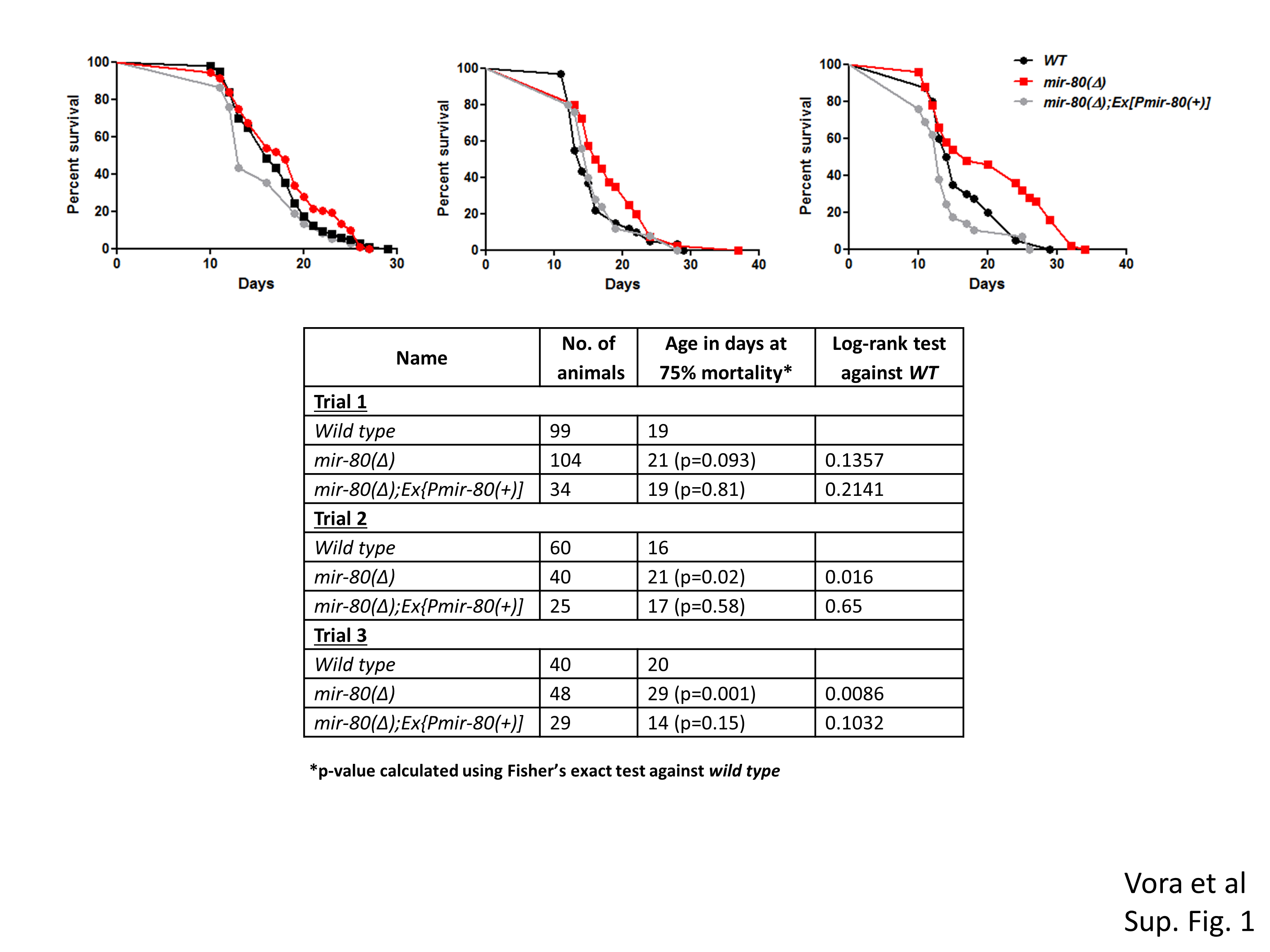

Supplement: Figure S1 — Individual lifespan data for lifespan analysis of mir-80(Δ). We grew age-synchronized WT (black), mir-80(Δ) (red) or the mir-80(+) (grey) under standard plate conditions (200C, OP50-1). At day 9, we placed 10 healthy animals per plate, and we scored viability as movement away from pick touch on the indicated days. Statistics were calculated using the OASIS software. Details are presented in Table format. Three additional trials that did not include the rescued strain, as well as other trials featured in the text, showed similar trends, on the order of 10–30% lifespan extension. Note the some trials with mir-80 transgene rescue suggest that overexpression of mir-80 may be deleterious, and that in a small minority of trials, we did not see life extension although the culture always trended in that direction. (TIF) [file pgen.1003737.s001.tif]

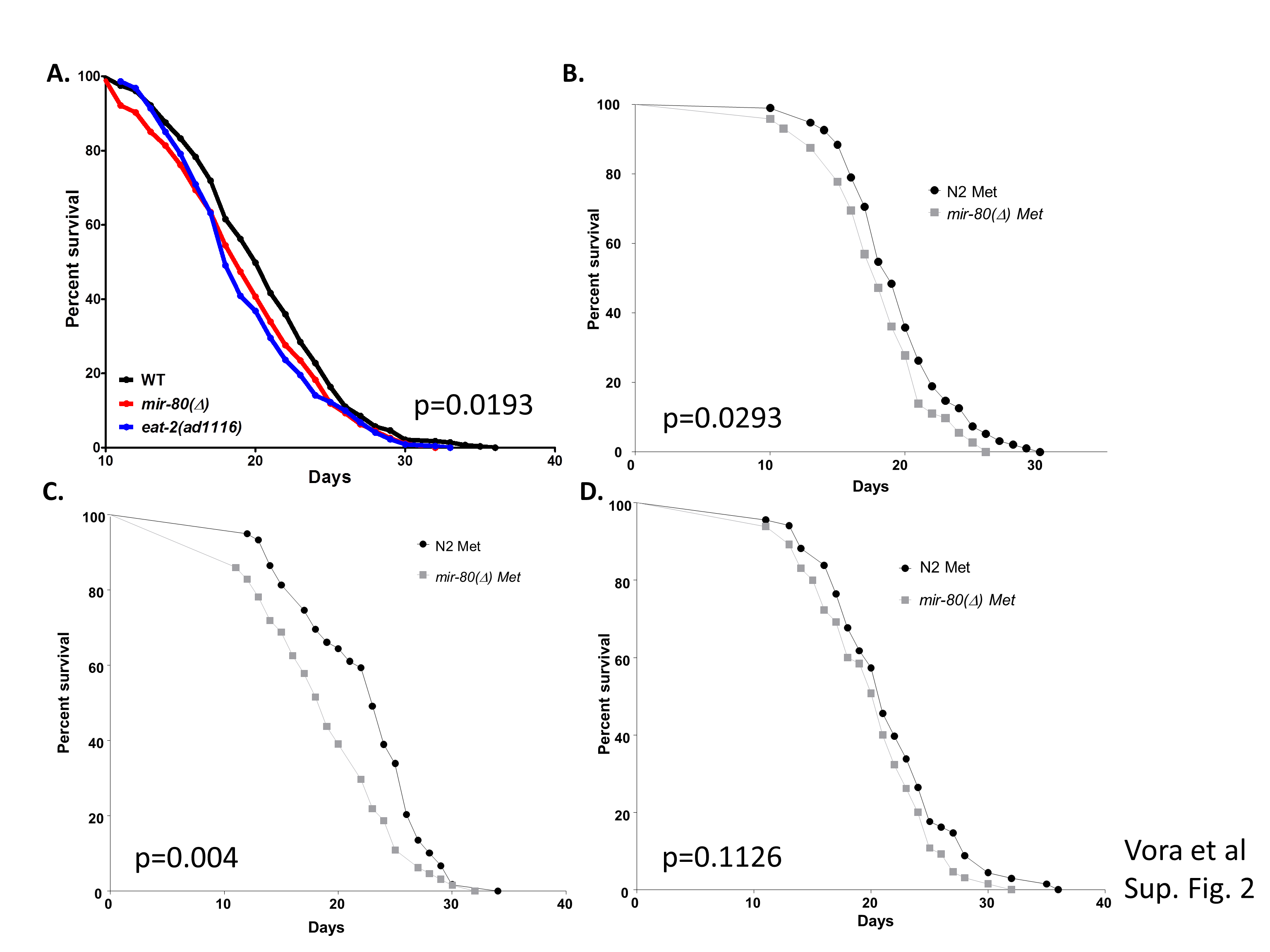

Supplement: Figure S2 — The mir-80(Δ) mutant exhibits hypersensitivity to the DR-mimetic drug metformin, similar to DR mutant eat-2. We grew age-synchronized WT (black), mir-80(Δ) (red) or the eat-2 mutant (blue) under standard plate conditions supplemented with 50 mM metformin (20°C, OP50-1). At day 9, we placed 10 healthy animals per plate, ≥40 per strain per trial, and we scored viability as movement away from pick touch on the indicated days. The upper left graph (A) represents data combined from 3 independent trials, which are presented individually in the other panels (B-D). Statistics are calculated using the Log-rank Test. Error bars indicate ± S.E.M. Metformin reduces lifespan for mir-80(Δ) and eat-2 as compared to WT. (TIF) [file pgen.1003737.s002.tif]

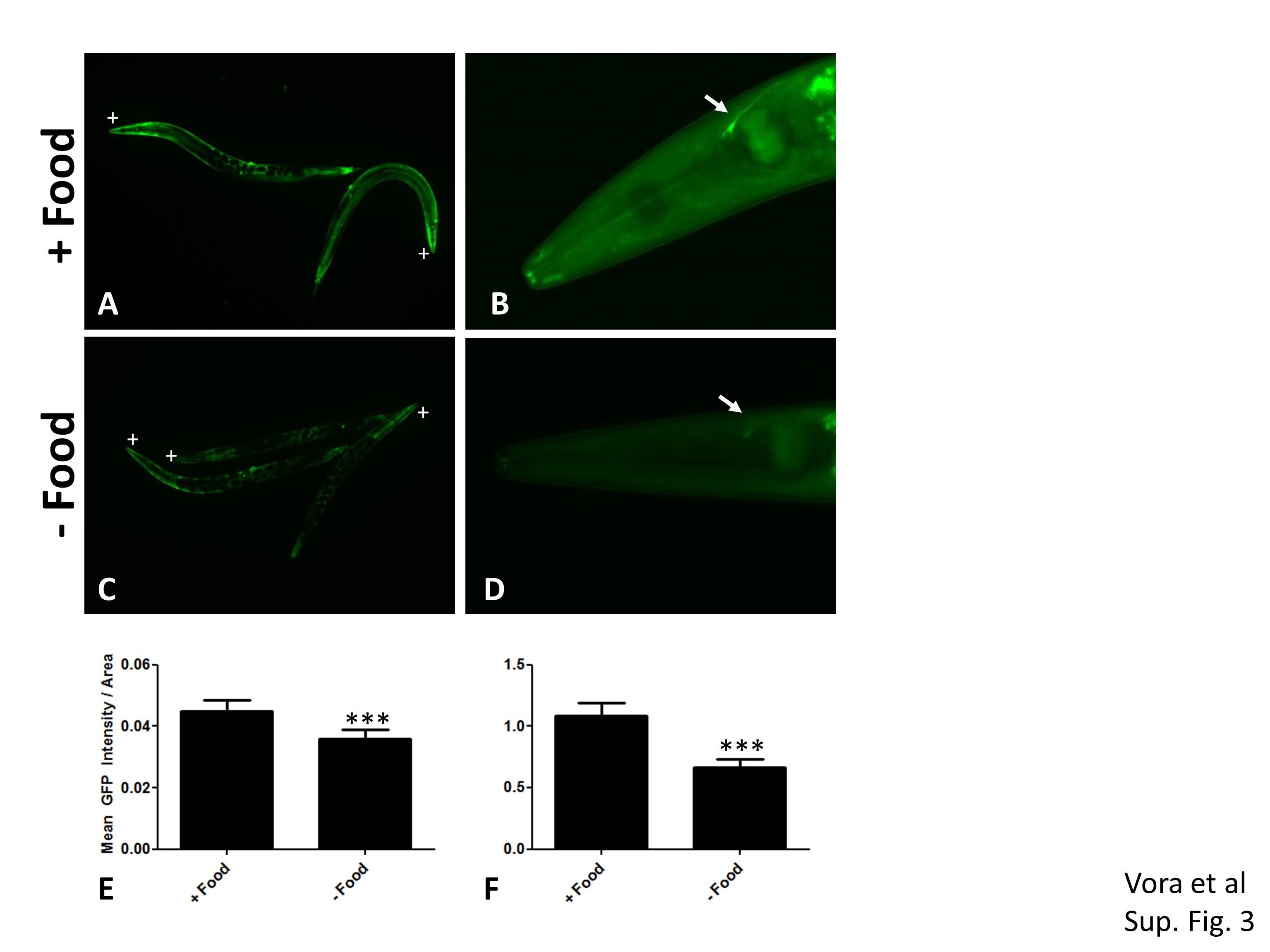

Supplement: Figure S3 — Published mir-80::GFP reporters are regulated by food availability. Fig. S3A. Example of expression in the Ex[Pmir-80::GFP] line VL211 ([17], wwEx18) grown in the presence of unlimited E. coli. Animals are at day 6 from the hatch for all images in this figure. + indicates anterior. Fig. S3B. Excretory duct cell in the Is[Pmir-80::GFP] in line VT1492 ([17], maIs196) grown in the presence of unlimited E. coli. White arrow indicates the fluorescent cell that is well labeled in this integrated line, tentatively identified as the excretory duct cell. . Example of expression of Ex[Pmir-80::GFP] in line VL211 grown in the presence of unlimited E. coli to young adulthood and then switched to no food for 48 hours. Fig. S3D. Example of Is[Pmir-80::GFP] line VT1492 excretory duct cell (white arrow) grown in the presence of unlimited E. coli to young adulthood and then switched to no food for 48 hours. Fig. S3 E,F. Quantitation of fluorescence signals for mir-80 promoter fusion reporter lines in food vs. food limitation. (E) Fluorescence of overall Ex[Pmir-80::GFP] line VL211, (F) excretory duct cell Is[Pmir-80::GFP] line VT1492; after 48 hrs on no-food plates. Food limitation in these studies was by dietary deprivation [5], but food dilution on solid NGM media [8] and food dilution in liquid media [37] induced similar changes in these lines (see Fig. S5). Graphs represent measured fluorescence levels (whole body for E; cell region for F) for at least 50 animals per DR regimen. Error bars represent mean intensity ± S.E.M. Pairwise comparisons were made using Two-tailed Students' T-test. *** - p<0.0005, ** - p<0.005. Same exposure times were used for complementary panels. (TIF) [file pgen.1003737.s003.tif]

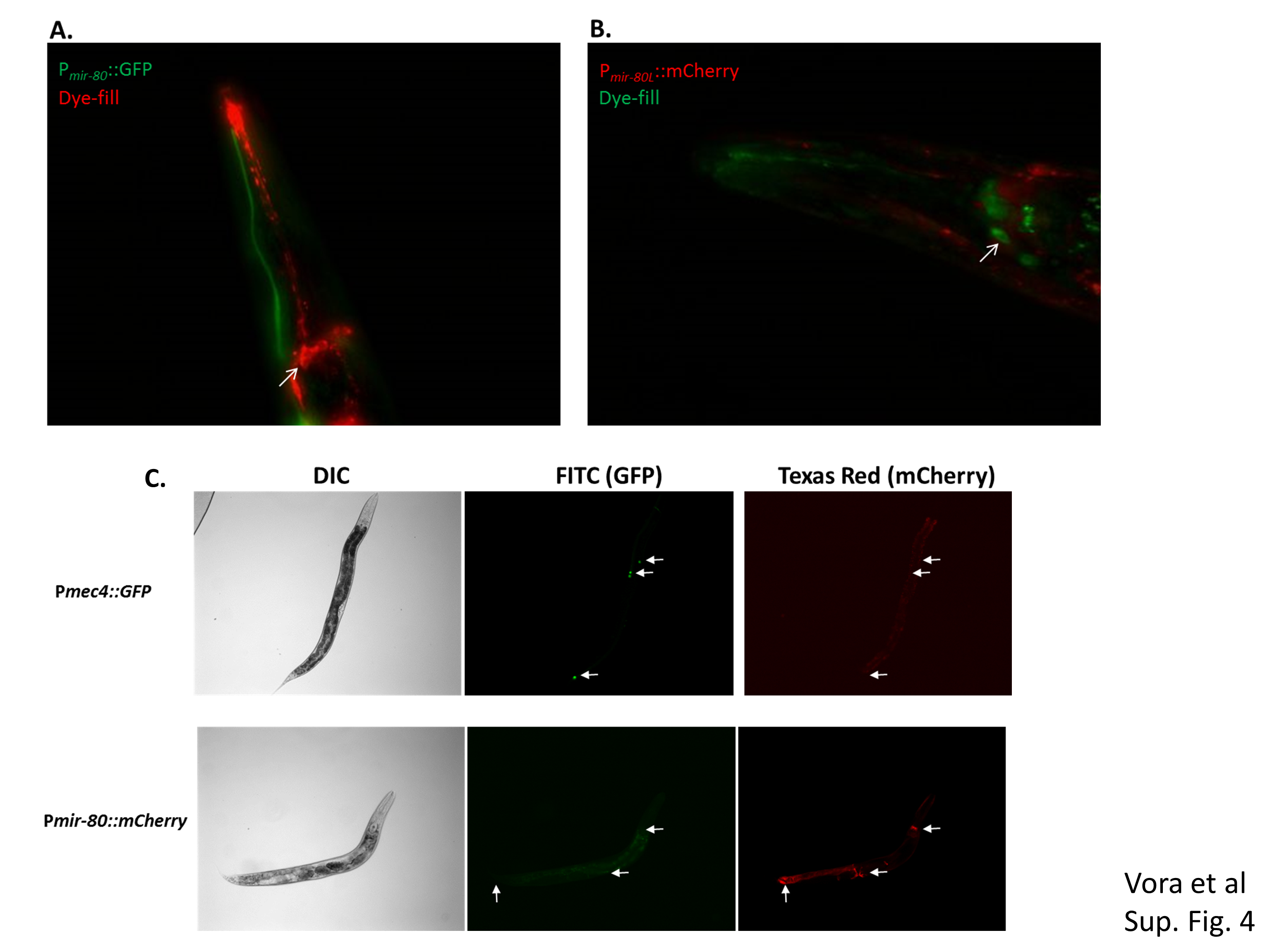

Supplement: Figure S4 — A,B. Two different transcriptional reporters for mir-80 fail to co-localize with ASI sensory neurons, so up-regulation of skn-1::GFP expression in those neurons in DR may be a non-autonomous consequence of miR-80 activity. The expression of transcription factor skn-1 in the two head ASI sensory neurons can be necessary for DR lifespan extension benefits [7], and we have shown that mir-80(Δ) increases the expression of a skn-1::GFP reporter in the ASI neurons (Fig. 2E). Thus, an important mechanistic question is whether miR-80 is present in the ASI neurons where it might cell-autonomously affect skn-1 expression. To test for mir-80 expression in ASI neurons, we took advantage of the fact that ASI neuron endings are open to the environment and can uptake fluorescent dye from their surroundings. We used a dye-filling assay to label the amphid sensory neurons in the Pmir-80 fluorescent reporter lines to test for co-expression (Pmir-80::GFP with red DiO, and Pmir-80L::mCherry with yellow DiI). We reared animals under standard conditions (ad lib OP50-1, 20°C). We labelled {A} VL211 expressing Pmir-80::GFP with red DiO, and reciprocally, {B} strain ZB3042 containing bzEx207[Pmir-80L::mCherry] with yellow fluorescent DiI, using a standard protocol that enables the amphid neurons that are open to the environment (ASI, ADL, ASK, AWB, ASH and, ASJ) to dye-fill. For both approaches, we never observed co-label of the mir-80 reporter with any amphid neurons (n = 30 per reporter); white arrows indicate ASI in representative images. Thus, although mir-80(Δ) influences skn-1::GFP expression in ASI neurons in older animals (Fig. 2E), expression data suggest miR-80 does not act cell autonomously in ASI neurons to exert this regulation. C. Absence of fluorescent bleed-through in through the GFP/DAPI filter sets in the Pmir-80L::mCherry lines Pmec-4::GFP (top row) and Pmir-80::mCherry (bottom row) lines were age-synchronized via alkaline bleaching and plated on standard NGM containing O [file pgen.1003737.s004.tif]

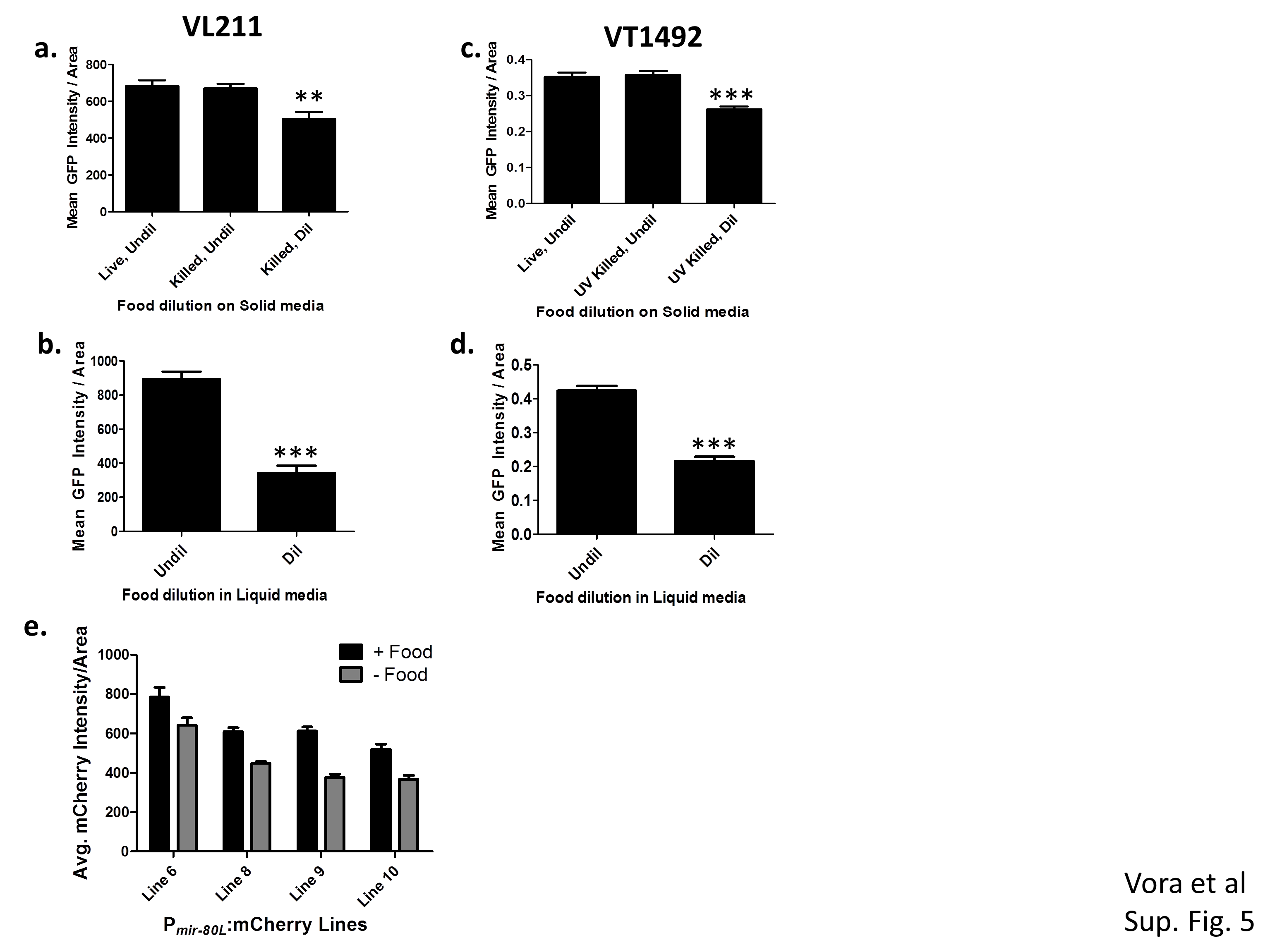

Supplement: Figure S5 — mir-80 is regulated by food availability under multiple food restriction conditions and as assayed with multiple reporters. We grew strains either under abundant food conditions or using the dietary limitation protocol indicated. Graphs represent measured fluorescence levels from areas indicated for 2 trials of at least 50 animals per DR regimen (except liquid DR for VL211, 17 worms as the strain bagged frequently), measured 48 hours after dietary limitation. Error bars represent Mean Intensity ± S.E.M. Pairwise comparisons were made using Two-tailed Students' T-test, ** - p<0.0005, ** - p<0.005. a) overall pmir-80GFP line VL211 expression, food dilution on solid NGM media. b) overall pmir-80GFP line VL211 expression, food dilution in liquid media. c) excretory duct cell pmir-80GFP line VT1492 expression, food dilution on solid NGM media. d) excretory duct cell pmir-80GFP line VT1492 expression, food dilution in liquid media. e) overall fluorescence pmir-80LmCherry expression for 4 independently derived lines, in abundant food or under dietary deprivation. In individual trials, 4/5 tested lines exhibited significant differences; one line was not regulated in this direction (not shown). (TIF) [file pgen.1003737.s005.tif]

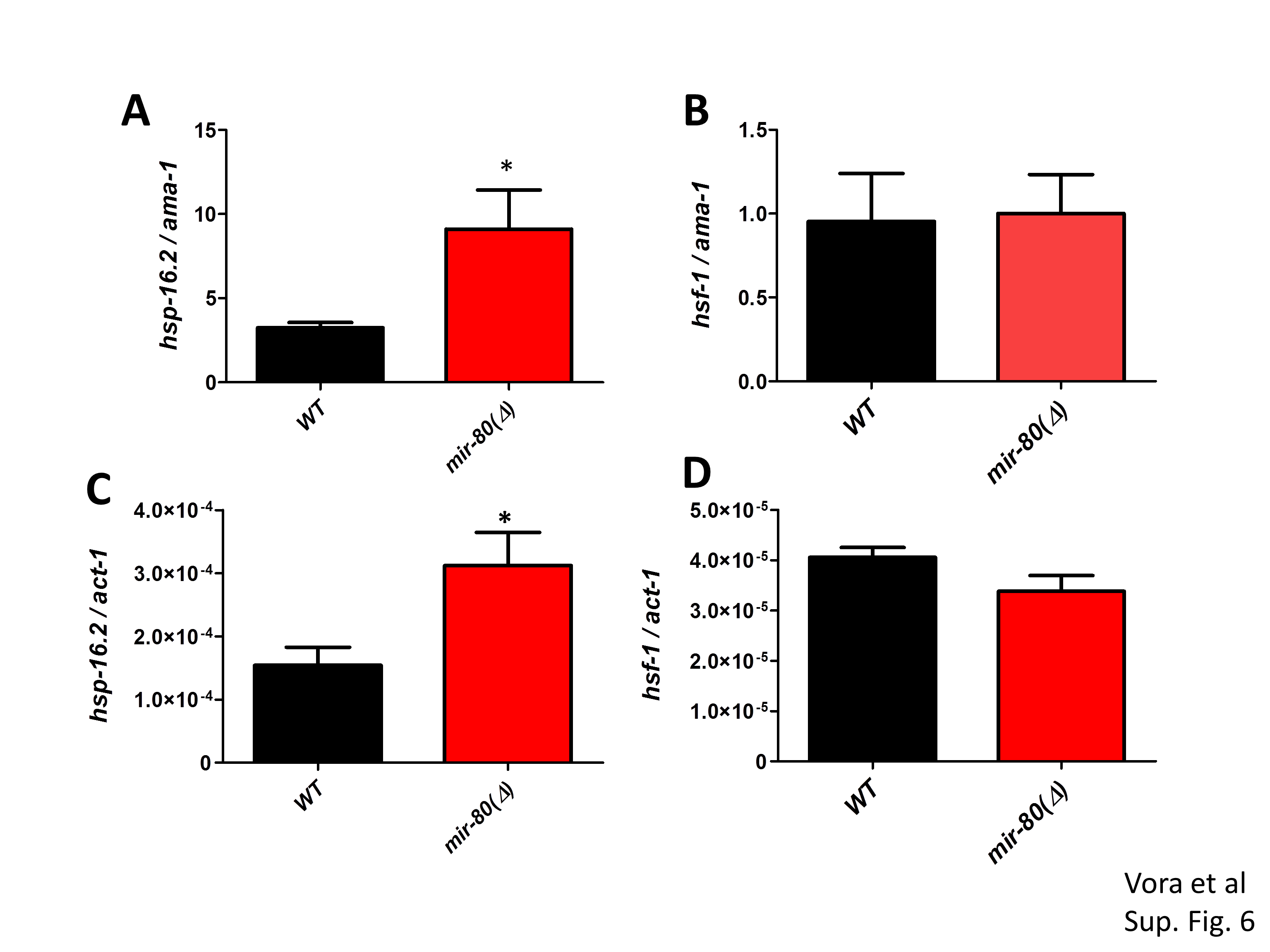

Supplement: Figure S6 — Relative HSF-1 target gene hsp-16.2 transcripts are elevated in mir-80(Δ), but HSF-1 transcript levels are maintained. Temperature-induced HSF-1 activation occurs post-translationally, via formation of an active trimer from inactive monomers that persist under basal conditions [60]. To address the question of whether mir-80(Δ) mutants exhibit increased HSF-1 transcriptional activity compared to WT, we used qRT-PCR to measure transcript levels of HSF-1 target gene hsp-16.2 [61], +/− mir-80. Our data suggest that loss of mir-80 indirectly upregulates HSF-1 activity to increase expression of HSF-1-dependent target genes. A. Transcriptional expression of HSF-1 target gene hsp-16.2 is elevated in mir-80(Δ). We grew age-synchronized WT and mir-80(Δ) under standard conditions of abundant food (20°C, OP50-1) and harvested animals at Day 4 for total RNA isolation. We normalized raw qPCR scores for hsp-16.2 (an hsf-1 target) to ama-1. Graphs represent cumulative data from 3 independent trials with 3 technical replicates per trial. Error bars represent ±S.E.M. for RAW transcript gene values normalized to RAW ama-1 levels. Normalized transcript levels of hsp-16.2 are elevated in mir-80(Δ), * p<0.1 (2-tailed Student's T-test). B. hsf-1 transcript levels (day 4) are not changed by the absence of miR-80. We grew age-synchronized WT animals in abundant food and harvested animals at Day 4 for RNA isolation. Raw qPCR scores for hsf-1 were normalized to ama-1. Graphs represent cumulative data from 3 independent trials with 3 technical replicates per trial. Error bars represent ±S.E.M for RAW transcript gene values normalized to RAW ama-1 levels. In Day 4 animals normalized hsf-1 transcript levels were similar in WT and mir-80(Δ) (p = 0.90; 2-tailed Student's T-test). We noted a trend toward lower hsf-1 transcript levels at day 7 (not shown), which we think reflects modulation of the dynamic transcriptional network altered by miR-80. C. Transcriptional expression of HSF-1 target gene h [file pgen.1003737.s006.tif]

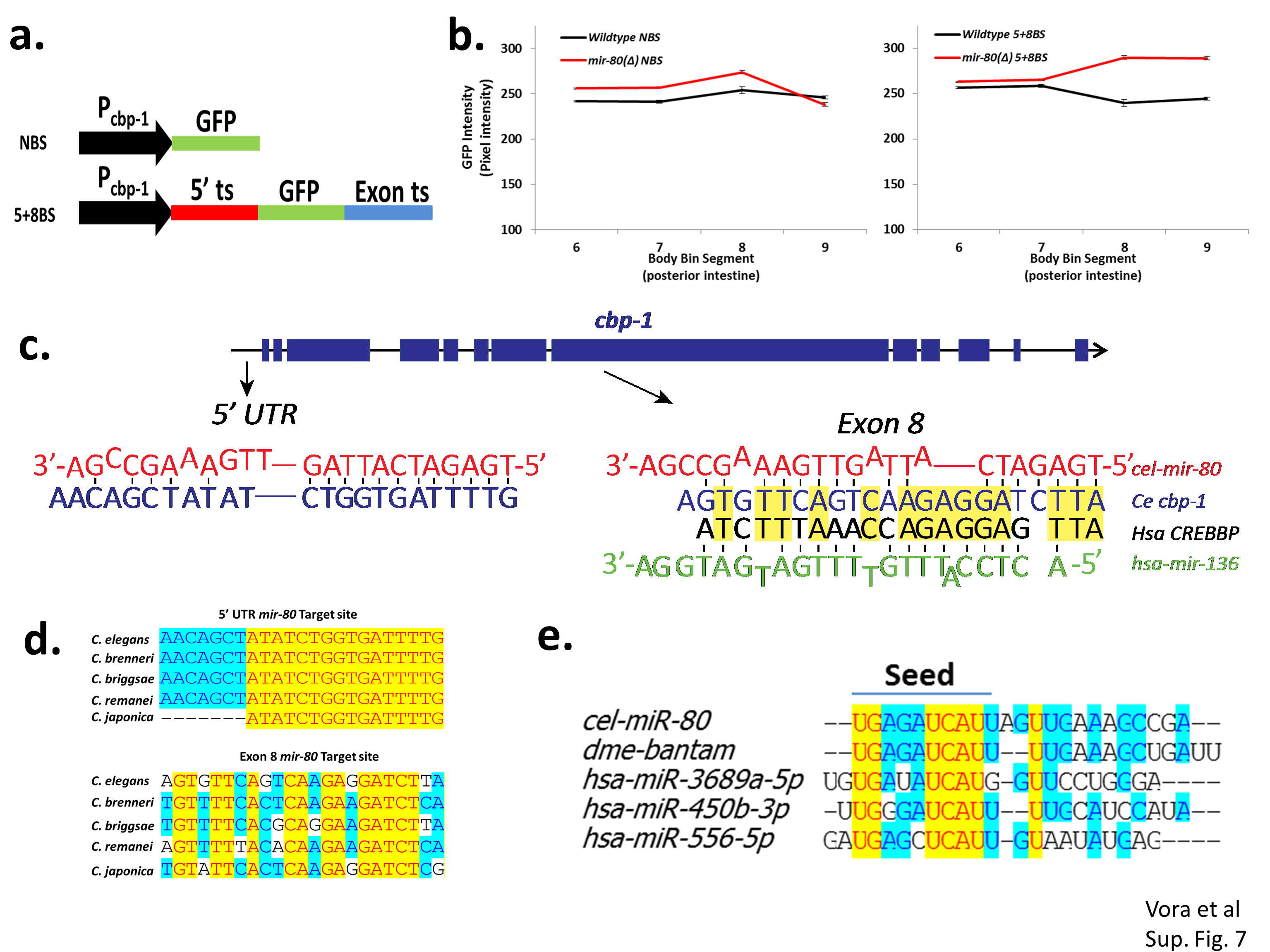

Supplement: Figure S7 — miR-80 may directly target cbp-1 mRNA in the posterior gut, is a conserved miRNA and potential binding sites for miRNAs in human CREBBP are present at sites analogous to those in C. elegans. A. Constructs used to test candidate miR-80 binding sites present in cbp-1 for roles in translational repression. We used the native cbp-1 promoter to express GFP reporters that included i) GFP lacking any candidate miR-80 binding sites (NBS); ii) the 5′ UTR candidate miR-80 binding site and the exon 8 candidate miR-80 binding site from cbp-1 (5+8BS). B. Shown are GFP intensities for extrachromosomal p cbp-1GFP constructs without candidate miR-80 binding sites (NBS, left panel) or with the 5′ and exon 8 candidate miR-80 binding sites added (5+8BS); in WT (black line) or mir-80(Δ) (red line) backgrounds, day 7, n>30, posterior gut. Animal segments imaged and fluorescence measured as in Fig. 3. The 5+8bs construct is expressed at a higher level when mir-80 is lacking, whereas the NBS construct is not. The 5+8BS high copy number construct also variably exhibited some “sponge” effects that might be attributed to titrating out endogenous miR-80 and family members (not shown). C. Exon structure of C. elegans cbp-1 is indicated by thick blue bars, introns in thin blue lines (see WormBase for details). The rna22 algorithm [62] predicts that miR-80 binds cbp-1 within the 5′ UTR and within exon 8. The potential alignments of miR-80 (red) to C. elegans cbp-1 (blue) sequences are indicated. Note that the seed match to the exon 8 region is a perfect 10 bp match for C. elegans, and that the target sequence is conserved to some degree in mouse and human CBP1. However, human miR-136 is even a better match in this region (shown here). D. The predicted miR-80 target sites are conserved in the various Caenorhabditis spp. E. Alignments of miR-80 family members from Drosophila melanogaster (bantam) and human with C. elegans miR-80. Seed region is indicated by black bar; yellow highlight, full conser [file pgen.1003737.s007.tif]
