# Supplementary material for: Deletion of microRNA-80 Activates Dietary Restriction to Extend C. elegans Healthspan and Lifespan
Source: PLoS Genet. 2013 Aug 29;9(8):e1003737. doi: 10.1371/journal.pgen.1003737 (PMC3757059; doi:10.1371/journal.pgen.1003737)
Supplement: Table S1 — An RNAi screen identifies hsf-1 and cbp-1 as required for the DR-associated Exmax shift phenotype in the mir-80(Δ) background. We examined the literature for genes experimentally implicated in DR and used RNAi to knock these genes down in the mir-80(Δ) background (Day 4, 20°C, three independent trials, 50 animals/trial). Since the Exmax shift is diagnostic of the DR state, we reasoned that genetic interventions that reversed the shift would define genes needed for the mir-80(Δ) DR pathway. P-values were calculated using two-tailed unpaired Students T-test; gene knockdowns that confer statistically significant p-values are in bold. (PDF) [file pgen.1003737.s008.pdf]

TABLE S1. An RNAi screen identifies *hsf-1* and *cbp-1* as required for the DR-associated Ex_{max} shift phenotype in the *mir-80(Δ)* background

We examined the literature for genes experimentally implicated in DR and used RNAi to knock these genes down in the *mir-80(Δ)* background (Day 4, 20°C, three independent trials, 50 animals / trial). Since the Ex_{max} shift is diagnostic of the DR state, we reasoned that genetic interventions that reversed the shift would define genes needed for the *mir-80(Δ)* DR pathway. P-values were calculated using two-tailed unpaired Students T-test; gene knockdowns that confer statistically significant p-values are in bold.

	***mir-80(Δ)* Ex_{max} Shifts**				
**DR genes**	**Mean wavelength**	**St. error**	**p-value against empty vector L4440**	**Basic Function**	**Required for**
Empty Vector	341.37	0.73			
aak-1	338.66	4.05	0.278	AMP-activated kinase; negatively regulates germline proliferation during dauer development [S1,S2]	hypothesized to induce DR like *aak-2* but not established [S1,S2]
aak-2	338.33	0.88	0.166	AMP-activated kinase; negatively regulates germline proliferation during dauer development [4]	Over-expression confers lifespan extension by solid and peptone dilution DR methods [4]
bec-1	342.00	0.00	0.770	essential autophagy gene [S3]	lifespan extension in *eat-2* mutant

					[S4]
cbp-1	**346.00**	**2.30**	**0.046**	histone acetyltransferase activity and required during embryogenesis for differentiation of all non-neuronal somatic cell types [34]	lifespan extension by three different DR regimens [34]
clk-1	341.33	1.76	0.984	biological timing abnormality; encodes a mitochondrial protein involved in ubiquinone biosynthesis [27]	lifespan extension by solid dilution DR method and *eat-2* mutant [4]
cup-4	342.33	0.88	0.657	encodes a coelomocyte-specific, ligand-gated ion channel [S4]	reduces lifespan in *eat-2* mutant [S4]
dve-1	343.66	0.88	0.293	forms complex for unfolded protein stress in mitochondria; vulval development [34]	lifespan extension by three different DR regimens [34]
hif-1	343.33	0.66	0.366	hypoxia-induced transcription factor complex; roles in oxygen homeostasis, tumor formation, glucose metabolism and inflammatory response [S5]	lifespan extension by solid DR method [S5]
hsf-1	**345.66**	**0.88**	**0.055**	heat shock factor; transcription regulator required for heat-shock and proteotoxicity response; [4,36]	required for lifespan extension by dietary deprivation DR method
ire-1	343.33	0.66	0.366	endoplasmic reticulum (ER) stress regulator; associated with lower levels of ER stress [S5]	required for lifespan and healthspan extension; lifespan extension by solid DR method [S5]
nlp-1	344.00	2.00	0.244	a neuropeptide that	none

				regulates acetylcholine-induced muscle contraction, expressed in neurons, pharyngeal and intestine [S6,S7]	documented, tested for regulation of pharyngeal contraction in DR mutants [S7]
rheb-1	341.33	0.66	0.984	mitochondrial unfolded protein response gene required for normal growth rates, body size, osmoregulation, reproduction, and locomotion [S8]	lifespan extension by alternate day or every two day fasting regime and dietary deprivation DR method [S8]
sir-2.1	342.33	2.33	0.671	encodes one of 4 *C. elegans* proteins that are HDACs that influence lifespan [S9]	extra-chromosomal transgenic animals with over-expression experience lifespan extension [S9]
skn-1	343.33	0.66	0.366	transcription factor required for embryonic development/EMS blastomere [7]	needed in ASI neurons for food limitation-induced DR lifespan benefits [7] and *eat-2* mutant
ubc-18	341.66	0.88	0.892	ubiquitin conjugating enzyme; regulates pharyngeal morphogenesis during early embryonic development [S10]	reduces lifespan in *eat-2* mutant [S10]
vps-34	344.66	1.76	0.144	vacuolar protein sorting in yeast, autophagy; regulates endocytosis required for growth and	required for *bec-1* pathway [S11]

				development [S11]	
