# Supplementary material for: Deletion of microRNA-80 Activates Dietary Restriction to Extend C. elegans Healthspan and Lifespan
Source: PLoS Genet. 2013 Aug 29;9(8):e1003737. doi: 10.1371/journal.pgen.1003737 (PMC3757059; doi:10.1371/journal.pgen.1003737)
Supplement: Table S2 — RNAi directed against known DR genes in the mir-80(Δ) background identify genes required for the low age pigment level phenotype. We used RNAi to knockdown genes previously implicated in DR in the mir-80(Δ) background (Day 4, 20°C, three independent trials, 50 animals/trial) and quantitated age pigment levels relative to endogenous Trp levels. Since low age pigment/Trp ratios (AGE/TRP) are associated with the DR state [19], we reasoned that genetic interventions that elevated age pigment levels would help identify genes needed for the mir-80(Δ) DR pathway and/or for enhanced healthspan. P-values were calculated using the Unpaired two-tailed Student's T-test, values within the 90% confidence level are bold, increase or decrease indicated by arrows. (PDF) [file pgen.1003737.s009.pdf]

TABLE S2. RNAi directed against known DR genes in the *mir-80(Δ)* background identify genes required for the low age pigment level phenotype.

We used RNAi to knockdown genes previously implicated in DR in the *mir-80(Δ)* background (Day 4, 20°C, three independent trials, 50 animals/trial) and quantitated age pigment levels relative to endogenous Trp levels. Since low age pigment/Trp ratios (AGE/TRP) are associated with the DR state [19], we reasoned that genetic interventions that elevated age pigment levels would help identify genes needed for the *mir-80(Δ)* DR pathway and/or for enhanced healthspan. P-values were calculated using the Unpaired two-tailed Student's T-test, values within the 90% confidence level are bold, increase or decrease indicated by arrows.

***mir-80(Δ)* Age Pigments**			
RNAi Clone	Mean (AGE/TRP)	St. Error	p-value against own L4440
**L4440 vector**	0.459	0.02	
aak-1	0.643	0.03	**0.04** ↑
aak-2	0.625	0.02	**0.06** ↑
bec-1	0.360	0.11	0.292
cbp-1	0.613	0.09	**0.099** ↑
clk-1	0.348	0.02	0.208
cup-4	0.414	0.01	0.603
dve-1	0.543	0.02	0.336
hif-1	0.539	0.15	0.420
hsf-1	0.651	0.08	**0.041** ↑
ire-1	0.381	0.03	0.374
nlp-1	0.400	0.06	0.511
pgp-2	0.419	0.02	0.649
rheb-1	0.352	0.01	0.223
sir-2.1	0.270	0.06	**0.041**
skn-1	0.683	0.02	**0.015** ↑

ubc-18	0.432	0.02	0.754
vps-34	0.599	0.05	0.121
