# Supplementary material for: Deletion of microRNA-80 Activates Dietary Restriction to Extend C. elegans Healthspan and Lifespan
Source: PLoS Genet. 2013 Aug 29;9(8):e1003737. doi: 10.1371/journal.pgen.1003737 (PMC3757059; doi:10.1371/journal.pgen.1003737)
Supplement: Table S3 — Strains used in this study. (PDF) [file pgen.1003737.s010.pdf]

Supplementary Table S3—Strains used in this study

Strain name	Genotype
ZB3035	*mir-80(nDf53)* 6x outcrossed to our N2 wild type
ZB3036	*mir-80(nDf53);nEx1457* (array nEx1457 from [18])
ZB3037	*mir-80(nDf53);is007* (transgene is007 from *[7])*
ZB3038	*eat-2(ad1116);is007* (transgene is007 from *[7])*
ZB3044	*mir-80(nDf53);daf-16(mgDf50)*
ZB3045	*mir-80(nDf53);hsf-1(sy441)(ts)*
ZB3039	*bzEx207*[pMV100($P_{mir\text{-}80L}$*:mCherry*) + pRF6{*rol-6(su1006)*}] Line #6
ZB3040	*bzEx208*[pMV100($P_{mir\text{-}80L}$*:mCherry*) + pRF6{*rol-6(su1006)*}] Line #7
ZB3041	*bzEx209*[pMV100($P_{mir\text{-}80L}$*:mCherry*) + pRF6{*rol-6(su1006)*}] Line #8
ZB3042	*bzEx210*[pMV100($P_{mir\text{-}80L}$*:mCherry*) + pRF6{*rol-6(su1006)*}] Line #9
ZB3043	*bzEx211*[pMV100($P_{mir\text{-}80L}$*:mCherry*) + pRF6{*rol-6(su1006)*}] Line #10
ZB3047	*bzEx213*[pMV101($P_{cbp\text{-}1}$*:GFP NBS*) + P_{mec4}*:mCherry*] Line #2
ZB3054	*bzEx220*[pMV102($P_{cbp\text{-}1}$*:5'bs-GFP-3'BS*) + P_{mec4}*:mCherry*] Line #2
ZB3056	*mir-80(nDf53); bzEx222*[pMV102($P_{cbp\text{-}1}$*:5'bs-GFP-3'BS*) + P_{mec4}*:mCherry*] Line #2
ZB3058	*mir-80(nDf53); bzEx224*[pMV101($P_{cbp\text{-}1}$*:GFP NBS*) + P_{mec4}*mCherry*] Line #2
ZB3060	*mir-80(nDf53)*;uIs57[$P_{unc\text{-}119:}$*sid-1*, $P_{unc\text{-}119:}$*yfp* + $P_{mec\text{-}6:}$*mec-6]* Transgene uIs57 is from *[59]*

TU3270	*uIs57*[$P_{unc\text{-}119:}$*sid-1, *$P_{unc\text{-}119:}$*yfp* + $P_{mec\text{-}6:}$*mec-6]* Transgene uIs57 is from *[59]*
