# Supplementary material for: Deletion of microRNA-80 Activates Dietary Restriction to Extend C. elegans Healthspan and Lifespan
Source: PLoS Genet. 2013 Aug 29;9(8):e1003737. doi: 10.1371/journal.pgen.1003737 (PMC3757059; doi:10.1371/journal.pgen.1003737)
Supplement: Table S4 — Summary of lifespan experiments. Median lifespan is calculated from the GraphPad Prism Software while Maximum lifespan was calculated by the mean lifespan of the oldest 10% cohort in each experiment. P-values are calculated from Log-Rank Tests for the entire population for the complete lifespan. (PDF) [file pgen.1003737.s011.pdf]

Supplementary Table S4:

Summary of lifespan experiments. Median lifespan is calculated from the GraphPad Prism Software while Maximum lifespan was calculated by the mean lifespan of the oldest 10% cohort in each experiment. P-values are calculated from Log-Rank Tests for the entire population for the complete lifespan.

**Trial 1 (Fig 1D)**	Number of animals	Median Lifespan (days)	Maximal Lifespan (days)	p-value against N2	% change against N2
N2	78	13.5	17.94		
Mir-80(nDf53)	81	14	21.22	p<0.01	18.2

**Trial 2 (Fig 1D)**	Number of animals	Median Lifespan (days)	Maximal Lifespan (days)	p-value against N2	% change against N2
N2	99	16	23.48		
mir-80(nDf53)	100	16.5	25.23	p<0.05	7.5

**Trial 3 (Fig 1D)**	Number of animals	Median Lifespan (days)	Maximal Lifespan (days)	p-value against N2	% change against N2
N2	98	16.5	23.87		
mir-80(nDf53)	99	17.0	25.05	p=0.5398	4.9

**Trial 4 (Fig 1D)**	Number of animals	Median Lifespan (days)	Maximal Lifespan (days)	p-value against N2	% change against N2
N2	99	16.0	23.66		
mir-80(nDf53)	104	18.0	26.45	p=0.1357	11.79
mir-80(nDf53);Ex[Pmir-80:mir-80]	37	13.0	24.5	p=0.40	3.5

**Trial 5 (Fig 1D)**	Number of animals	Median Lifespan (days)	Maximal Lifespan (days)	p-value against N2	% change against N2
N2	60	14	26.27		
mir-80(nDf53)	40	16.5	27.76	p<0.01	5.6
mir-80(nDf53);Ex[Pmir-80]	25	15	21.76	p=0.65	-17.1

**Trial 6 (Fig 1D)**	Number of animals	Median Lifespan (days)	Maximal Lifespan (days)	p-value against N2	% change against N2
N2	40	14.5	24.56		
mir-80(nDf53)	50	17	33.1	p<0.01	34.7
mir-80(nDf53);Ex[Pmir-80]	29	13.00	23.8	p<0.05	-3.09

**Trial 1 (Fig 4c)**	Number of animals	Median Lifespan (days)	Maximal Lifespan (days)	p-value against N2	% change against N2
N2	58	19	29.36		
mir-80(nDf53)	60	21	28.83		-8.00
daf-16(mgDf50)	61	13.00	20.6	p<0.05	-29.83
mir-80(nDf53);daf-16(mgDf50)	57	14	20.44	p<0.05	-30.38

**Trial 2 (Fig 4c)**	Number of animals	Median Lifespan (days)	Maximal Lifespan (days)	p-value against N2	% change against N2
N2	63	17	26.82		
mir-80(nDf53)	60	21	34.25	p<0.01	27.7
daf-16(mgDf50)	59	14	22.44	p<0.05	-16.38
mir-80(nDf53);daf-16(mgDf50)	60	16	24.15	p<0.05	-10.02

**Trial 1 (Fig 4d)**	Number of animals	Median Lifespan (days)	Maximal Lifespan (days)	p-value against N2 (L4440)	% change against N2 (L4440)
N2;L4440	93	14	29.94		
mir-80(nDf53);L4440	83	17	31.61	p<0.01	5.5
N2(daf-2 RNAi)	80	18	38.56	p<0.01	28.79
mir-	79	17	36.97	p<0.01	23.4

80(nDf53;daf-2 RNAi					

**Trial 2 (Fig 4d)**	Number of animals	Median Lifespan (days)	Maximal Lifespan (days)	p-value against N2 (L4440)	% change against N2 (L4440)
N2;L4440	50	18	34.1		
mir-80(nDf53);L4440	30	20	39.8	p<0.01	16.7
N2(daf-2 RNAi)	48	27	44.05	p<0.01	29.1
mir-80(nDf53;daf-2 RNAi	42	27	36.5	p<0.01	7.0

**Trial 3 (Fig 4d)**	Number of animals	Median Lifespan (days)	Maximal Lifespan (days)	p-value against N2 (L4440)	% change against N2 (L4440)
N2;L4440	50	18	26.57		
mir-80(nDf53);L4440	31	18	34.75	p<0.01	30.7
N2(daf-2 RNAi)	50	22	40.67	p<0.01	53.06
mir-80(nDf53;daf-2 RNAi	41	24	42.09	p<0.01	58.4

**Trial 1 (Fig 6c)**	Number of animals	Median Lifespan (days)	Maximal Lifespan (days)	p-value against N2 (L4440)	% change against N2 (L4440)
N2;L4440	50	18	26.57		
mir-80(nDf53);L4440	31	18	34.75	p<0.01	30.7
N2(cbp-1 RNAi)	42	16	20.12	p<0.01	-24.27
mir-80(nDf53);cbp-1 RNAi	40	16	20.54	p<0.01	-22.69

**Trial 2 (Fig 6c)**	Number of	Median	Maximal	p-value	%

	animals	Lifespan (days)	Lifespan (days)	against N2 (L4440)	change against N2 (L4440)
N2(cbp-1 RNAi)	40	18	18.87	NA	NA
mir-80(nDf53);cbp-1 RNAi	39	18	19.09	NA	NA

NA = Wildtype and mir-80(Δ) not performed for this trial.

**Trial 3 (Fig 6c)**	Number of animals	Median Lifespan (days)	Maximal Lifespan (days)	p-value against N2 (L4440)	% change against N2 (L4440)
N2(cbp-1 RNAi)	60	13	19.8	NA	NA
mir-80(nDf53);cbp-1 RNAi	60	15	20.13	NA	NA

NA = Wildtype and mir-80(Δ) not performed for this trial.

Individual Trials for Age pigment accumulation at Day 4 for Figs 4a, 5a, and 6a.

**Fig 4,5,6a**	**Trial 1**	**Trial 2**	**Trial 3**	**Mean**	**SEM**	**T-test**
**WT**	346	347	348	347	0.57	
mir-80(Δ)	344	340	342	342	1.15	p<0.05 (vs WT)
daf-16(mgDf50)	347	349	349	348.33	0.67	NS (vs WT)
mir-80(Δ); daf-16(mgDf50)	344	350	347	347	1.73	NS (vs WT)
**WT (Empty vector)**	345	346	348	346.3	0.88	
***mir-80(Δ)* (Empty Vector)**	342	342	340	341.67	0.67	p<0.001 (aginst wt EV)
***mir-80(Δ)* (*hsf-1 RNAi*)**	347	346	344	345.67	0.88	p=0.055 (against *mir-80(Δ) EV)*
***mir-80(Δ)* (*cbp-1 RNAi*)**	346	342	350	346	2.3	p<0.05 (against *mir-80(Δ) EV)*

Individual Trials for Age pigment accumulation at Day 4 for Figs 4b, 5b, and 6b.

**Fig 4,5,6b**	**Trial 1**	**Trial 2**	**Trial 3**	**Mean**	**SEM**	**T-test**

**WT**	0.50	0.48	0.57	0.51	0.02	
mir-80(Δ)	0.30	0.36	0.30	0.32	0.02	$p<0.05$ (vs WT)
daf-16(mgDf50)	1.03	1.15	1.59	1.25	0.16	$p<0.01$ (vs WT)
mir-80(Δ); daf-16(mgDf50)	0.99	1.23	1.51	1.24	0.14	$p<0.01$ (vs WT)
**WT (Empty vector)**	1.01	0.75	0.799	0.85	0.08	
***mir-80(Δ)* (Empty Vector)**	0.36	0.74	0.36	0.49	0.12	$p<0.001$ (aginst wt EV)
***mir-80(Δ)* (*hsf-1 RNAi*)**	0.67	0.494	0.789	0.65	0.08	$p<0.05$ (against *mir-80(Δ) EV)*
***mir-80(Δ)* (*cbp-1 RNAi*)**	0.54	0.49	0.79	0.60	0.09	$p<0.05$ (against *mir-80(Δ) EV)*
