# Supplementary material for: Deletion of microRNA-80 Activates Dietary Restriction to Extend C. elegans Healthspan and Lifespan
Source: PLoS Genet. 2013 Aug 29;9(8):e1003737. doi: 10.1371/journal.pgen.1003737 (PMC3757059; doi:10.1371/journal.pgen.1003737)
Supplement: Text S1 — Supplementary methods. (PDF) [file pgen.1003737.s012.pdf]

Supplemental Methods

Vora et al.

Measurement of pharyngeal pumping rates

We assayed age-synchronized animals (n≥10, 3 independent trials) for pharyngeal pumping at Day 4, Day 11 (post-hatching) (20^0C, OP50-1), scoring pumps for 30 seconds for healthy animals [1,2] under a standard dissecting microscope. Data were compared using the One-way ANOVA followed by Newman-Keuls multiple comparison test.

Dye-filling of amphid neurons to identify colocalization with ASI neurons

We raised age synchronized animals (n≥30, 3 independent trials) carrying a transcriptional reporter for *mir-80* [extrachromosomal high copy number line (Strain VL211) [3] and ZB3039, ZB3040, ZB3041, ZB3042, ZB3043, which are independent lines of $P_{mir\text{-}80L}$mCherry (see Supplementary Table S3) under standard conditions (20^0C, OP50-1, NGM plates) from Day 1 to Day 4. On Day 4, animals were placed in 1 ml of M9 buffer containing 2.5 ug/ml of DiI or DiO. Tubes were inverted several times and incubated for 30 mins – 1 hour in the dark. Worms were centrifuged at 2000 rpm for 1 min and supernatant was replaced with fresh M9. This was repeated three times. After final wash, worms were transferred to a seeded NGM plate and incubated in the dark for 30-90 mins for destaining and imaging.

P$_{mir-80}$*GFP* analysis for different DR regimens

Dietary Deprivation

We raised age synchronized animals (n≥30, 3 independent trials) carrying a transcriptional reporter for *mir-80* [integrated low copy number line (Strain VT1492), extrachromosomal high copy number line (Strain VL211) [3] and ZB3039, ZB3040, ZB3041, ZB3042, ZB3043, which are independent lines of *P$_{mir-80L}$*mCherry (see Supplementary Table S3) under standard conditions (20^{0}C, OP50-1, NGM plates) from Day 1 to Day 4. On Day 4, we moved control animals to plates with fresh OP50-1and experimental animals to dietary deprivation (DD plates, containing no OP50-1 and 100 ug/ml Ampicillin, 50 uM FUdR). We took images 2 days after transfer to plates lacking food, at Day 7 after the hatch. For VT1492, we measured signals using ImageJ with a region-of-interest (ROI) centered around the single cell in the head. For VL211 and ZB3039-3042, we quantified signals using the ImageJ software with a ROI defined around the entire body of animal, dividing total intensity by the area of the animal body.

Liquid DR

We raised age-synchronized animals (n≥30, 3 independent trials) carrying a *mir-80* transcriptional reporter (integrated low copy number line (Strain VT1492) and extrachromosomal high copy number line (Strain VL211)) [3] under standard conditions (20^{0}C, OP50-1, NGM plates) from Day 1 to Day 4. On day 4, we moved control animals to 1 ml S-basal liquid medium containing 5 ml pelleted OP50-1 cells from overnight growth in LB medium; we moved experimental animals to 1 ml S-basal liquid medium containing 1:100 diluted 5 ml pelleted OP50-1 cells from overnight growth medium in LB medium. We then reared animals at 20^{0}C in 24-well plates with gentle shaking and

moved animals to fresh S-basal medium (diluted or undiluted) every 24 hours until imaging on Day 7. For VT1492, we measured fluorescence using ImageJ with a region-of-interest (ROI) centered around the single cell in the head; for VL211, we measured signals with an ROI defined around the entire body of animal and divided total intensity by the body area.

DR by UV-killed Diluted bacteria on solid media

We raised age-synchronized animals (n≥30, 3 independent trials) carrying a transcriptional reporter for *mir-80* (integrated low copy number line (Strain VT1492) and extrachromosomal high copy number line (Strain VL211) [3] (20^0C, OP50-1, NGM plates) from Day 1 to Day 4. On Day 4, we moved control animals to standard NGM plates containing 150 ul of 5 ml of overnight culture of OP50-1 grown in LB medium; and moved experimental animals to standard NGM plates containing 150 ul of 1:1000 diluted overnight culture of OP50-1 grown in LB medium. Bacteria on both of these plates were previously killed using 2×10^6 uJ at 254 nm using the Stratagene UV Stratalinker 1800 instrument. We then reared animals under standard conditions (20^0C) and moved to fresh plates (killed diluted/undiluted) every 24 hours until imaging on Day 7. For VT1492, we measured signals using ImageJ with a region-of-interest (ROI) centered around the single cell in the head; for VL211, we scored an ROI defined around the entire body of animal and divided total intensity by the area of the animal body. For Figure 3D, we used the ImageJ program to create a 25 pixel segmented line covering the animal and we measured mean fluorescence intensity along the body. We divided the length into 12 equal bins and plotted the mean fluorescence intensity at each point.

RNAi experiments for measurement of age pigments and lifespan

We obtained RNAi clones for listed genes from the RNAi feeding library described in [4]. For lifespan assays, we synchronized strains by alkaline bleaching [5] and placed L1 larvae (Day 1) on RNAi plates (1mM IPTG) seeded with either control (L4440 empty vector) or gene-specific RNAi bacteria. We reared animals at 20^{0}C and moved these to fresh plates until Day 9; we used a minimum of 30 animals for lifespan assay. We counted survival at each time point until death of all animals and analyzed lifespan curves using the Log-Rank test using the Graphpad Prism 5 Software [6]. For age pigment analysis, we placed synchronized L1 larvae (day 1) on RNAi plates (1mM IPTG) seeded with either control (L4440 empty vector) or gene-specific RNAi bacteria. We reared animals at 20^{0}C and measured age pigments at Day 4 post-hatching.

Small RNA sequencing

Small RNAs were isolated using the mirVana Isolation Kit (Invitrogen). DNA libraries were prepared using the method that enriches for miRNAs, as previously described [7]. 4 nt barcodes were used to enable pooling of multiple libraries for Illumina GAIIx sequencing [8]. The Illumina sequencing tags were processed to remove the barcodes and 3' linker sequence, and then were aligned to the C. elegans microRNA precursor sequences provided by the miRBase Sequence Database (Release 19) [http://www.mirbase.org/] [9]. A total of 858,390 and 546,065 miRNA tags were obtained for the food and no-food samples, respectively. The number of miR-80 reads for the food and no-food samples were 158,048 and 66,879, respectively. A Bayesian approach was used to determine P-values for miRNA abundance levels as described in [10].
