# Supplementary material for: Deletion of microRNA-80 Activates Dietary Restriction to Extend C. elegans Healthspan and Lifespan
Source: PLoS Genet. 2013 Aug 29;9(8):e1003737. doi: 10.1371/journal.pgen.1003737 (PMC3757059; doi:10.1371/journal.pgen.1003737)
Supplement: Text S2 — References used exclusively in the supplemental information. (PDF) [file pgen.1003737.s013.pdf]

Supplemental References:

1. Apfeld J, O'Connor G, McDonagh T, DiStefano PS, Curtis R (2004) The AMP-activated protein kinase AAK-2 links energy levels and insulin-like signals to lifespan in *C. elegans*. Genes & Dev 18: 3004-3009.
2. Wormbase (2011) WormBase web site, http://www.wormbase.org, release WS224 date 8/17/2011.
3. Jia K, Levine B (2007) Autophagy is required for dietary restriction-mediated life span extension in C. elegans. Autophagy 3: 597-599.
4. Park SK, Link CD, Johnson TE (2010) Life-span extension by dietary restriction is mediated by NLP-7 signaling and coelomocyte endocytosis in C. elegans. FASEB J 24: 383-392.
5. Chen D, Thomas EL, Kapahi P (2009) HIF-1 modulates dietary restriction-mediated lifespan extension via IRE-1 in Caenorhabditis elegans. PLoS Genet 5: e1000486.
6. Chalasani SH, Kato S, Albrecht DR, Nakagawa T, Abbott LF, et al. (2010) Neuropeptide feedback modifies odor-evoked dynamics in Caenorhabditis elegans olfactory neurons. Nat Neurosci 13: 615-621.
7. Papaioannou S, Holden-Dye L, Walker RJ (2008) The actions of Caenorhabditis elegans neuropeptide-like peptides (NLPs) on body wall muscle of Ascaris suum and pharyngeal muscle of C. elegans. Acta Biol Hung 59 Suppl: 189-197.
8. Honjoh S, Yamamoto T, Uno M, Nishida E (2009) Signalling through RHEB-1 mediates intermittent fasting-induced longevity in C. elegans. Nature 457: 726-730.
9. Tissenbaum HA, Guarente L (2001) Increased dosage of a *sir-2* gene extends lifespan in *Caenorhabditis elegans*. Nature 410: 227-230.
10. Carrano AC, Liu Z, Dillin A, Hunter T (2009) A conserved ubiquitination pathway determines longevity in response to diet restriction. Nature 460: 396-399.
11. Furuya N, Yu J, Byfield M, Pattingre S, Levine B (2005) The evolutionarily conserved domain of Beclin 1 is required for Vps34 binding, autophagy and tumor suppressor function. Autophagy 1: 46-52.
